# PhcQ mainly contributes to the regulation of quorum sensing‐dependent genes, in which PhcR is partially involved, in *Ralstonia pseudosolanacearum* strain OE1‐1

**DOI:** 10.1111/mpp.13124

**Published:** 2021-08-21

**Authors:** Chika Takemura, Wakana Senuma, Kazusa Hayashi, Ayaka Minami, Yuki Terazawa, Chisaki Kaneoka, Megumi Sakata, Min Chen, Yong Zhang, Tatsuya Nobori, Masanao Sato, Akinori Kiba, Kouhei Ohnishi, Kenichi Tsuda, Kenji Kai, Yasufumi Hikichi

**Affiliations:** ^1^ Faculty of Agriculture and Marine Science Kochi University Nankoku Japan; ^2^ Graduate School of Life and Environmental Sciences Osaka Prefecture University Sakai Japan; ^3^ College of Resources and Environment Southwest University Chongqing China; ^4^ Interdisciplinary Research Center for Agriculture Green Development in Yangtze River Basin Southwest University Chongqing China; ^5^ Salk Institute for Biological Studies La Jolla California USA; ^6^ Graduate School of Agriculture Hokkaido University Sapporo Japan; ^7^ State Key Laboratory of Agricultural Microbiology, Interdisciplinary Sciences Research Institute, College of Plant Science and Technology Huazhong Agricultural University Wuhan China; ^8^ Present address: Central Research Institute Ishihara Sangyo Kaisha, LTD. Kusatsu Shiga Japan; ^9^ Present address: Agriculture Research Center Kochi Prefectural Nankoku Japan

**Keywords:** PhcQ, PhcR, quorum sensing, *Ralstonia pseudosolanacearum*, virulence

## Abstract

The gram‐negative plant‐pathogenic β‐proteobacterium *Ralstonia pseudosolanacearum* strain OE1‐1 produces methyl 3‐hydroxymyristate as a quorum sensing (QS) signal via the methyltransferase PhcB and senses the chemical through the sensor histidine kinase PhcS. This leads to functionalization of the LysR family transcriptional regulator PhcA, regulating QS‐dependent genes responsible for the QS‐dependent phenotypes including virulence. The *phc* operon consists of *phcB*, *phcS*, *phcR*, and *phcQ*, with the latter two encoding regulator proteins with a receiver domain and a histidine kinase domain and with a receiver domain, respectively. To elucidate the function of PhcR and PhcQ in the regulation of QS‐dependent genes, we generated *phcR*‐deletion and *phcQ*‐deletion mutants. Though the QS‐dependent phenotypes of the *phcR*‐deletion mutant were largely unchanged, deletion of *phcQ* led to a significant change in the QS‐dependent phenotypes. Transcriptome analysis coupled with quantitative reverse transcription‐PCR and RNA‐sequencing revealed that *phcB*, *phcK*, and *phcA* in the *phcR*‐deletion and *phcQ*‐deletion mutants were expressed at similar levels as in strain OE1‐1. Compared with strain OE1‐1, expression of 22.9% and 26.4% of positively and negatively QS‐dependent genes, respectively, was significantly altered in the *phcR*‐deletion mutant. However, expression of 96.8% and 66.9% of positively and negatively QS‐dependent genes, respectively, was significantly altered in the *phcQ*‐deletion mutant. Furthermore, a strong positive correlation of expression of these QS‐dependent genes was observed between the *phcQ*‐deletion and *phcA‐*deletion mutants. Our results indicate that PhcQ mainly contributes to the regulation of QS‐dependent genes, in which PhcR is partially involved.

## Graphical abstract

The regulators PhcQ and PhcR are mainly and partially, respectively, required for the regulation of quorum sensing‐dependent genes by the LysR family transcriptional regulator PhcA in *Ralstonia pseudosolanacearum* strain OE1‐1.

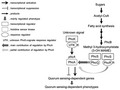

## 1 INTRODUCTION

Quorum sensing (QS) allows bacterial cells to communicate for the cooperative regulation of physiological processes coordinating various bacterial community activities (Ham, 2013; Waters & Bassler, 2005). To recognize their own populations, bacterial cells produce and secrete QS signals, which are small, diffusible molecules. Bacteria monitor QS signals to track changes in their cell numbers and to activate QS for the synchronous control of the expression of genes beneficial for vigorous replication and adaptation to environmental conditions, including virulence, such as the formation of biofilms and the production of virulence factors (Galloway et al., 2011; Rutherford & Bassler, 2012).

The gram‐negative plant‐pathogenic β‐proteobacterium *Ralstonia solanacearum* species complex (RSSC; Robinson et al., 2010) is globally distributed under diverse environmental conditions; strains infect more than 250 plant species in over 50 families and cause a potentially devasting bacterial wilt disease that seriously affects plant production worldwide (Mansfield et al., 2012). The RSSC is composed of four phylotypes (Fegan & Prior, 2006) and assigned to three distinct species: *Ralstonia pseudosolanacearum* (phylotypes I and III), *R*. *solanacearum* (phylotype II), and *Ralstonia syzygii* (phylotype IV) (Safni et al., 2014).

RSSC strains first invade the intercellular spaces of plant roots (Araud‐Razou et al., 1998; Hikichi et al., 2017; Vasse et al., 1995). After invading the roots, the bacterial cells attach to the surface of host cells, where they avoid plant innate immune responses (Genin & Denny, 2012; Hikichi et al., 2017; Kiba et al., 2018, 2020; Nakano et al., 2013) and grow vigorously to activate QS (Hikichi et al., 2017). The bacteria then invade xylem vessels, with the bacteria systemically spreading and multiplying throughout the xylem to activate QS (Genin & Denny, 2012; Hikichi et al., 2017; Vasse et al., 1995). This bacterial proliferation in the xylem leads to wilting symptoms in infected tomato plants. QS‐deficient mutants lose their virulence and their ability to invade xylem vessels (Genin & Denny, 2012; Hayashi et al., 2019a; Hikichi et al., 2017; Schell, 2000; Senuma et al., 2020). In addition, QS is thought to be conserved in all RSSC strains and is thus a required network for RSSC virulence (Castillo & Agathos, 2019; Genin & Denny, 2012).

*Ralstonia* has developed a genus‐specific QS system consisting of Phc QS cascade regulatory elements that respond to a unique fatty acid derivative signal (Flavier et al., 1997). Each strain of the RSSC produces either methyl 3‐hydroxypalmitate (3‐OH PAME) or methyl 3‐hydroxymyristate (3‐OH MAME) as a QS signal (Flavier et al., 1997; Kai et al., 2015; Schell, 2000; Ujita et al., 2019). RSSC strains synthesize the QS signal by the methyltransferase PhcB and sense the chemical through the sensor histidine kinase PhcS, activating QS (Genin & Denny, 2012; Kai et al., 2015; Schell, 2000; Ujita et al., 2019). The sensor histidine kinase PhcK is required for full expression of *phcA*, which encodes the LysR family transcriptional regulator PhcA, independently of QS signal production of PhcB (Senuma et al., 2020). In the active state of QS, PhcA activated through QS signal sensing of PhcS regulates QS‐dependent genes responsible for QS‐dependent phenotypes including virulence (Genin & Denny, 2012). This process leads to the induction of the production of ralfuranones, which are aryl‐furanone secondary metabolites, and the major exopolysaccharide EPS I, which is involved in the virulence of RSSC strains (Genin & Denny, 2012; Kai et al., 2014, 2015; Pauly et al., 2013; Schell, 2000; Wackler et al., 2011). Furthermore, these secondary metabolites are also associated with the feedback loop of QS‐dependent gene regulation (Hayashi et al., 2019b; Mori et al., 2018).

The *phc* operon consists of *phcB* and *phcS*, which encode Phc QS cascade regulatory elements, along with *phcR* and *phcQ* (Clough et al., 1997; Salanoubat et al., 2002; Tang et al., 2020). PhcR is an intracellular soluble two‐component protein composed of a sensor histidine kinase domain and a receiver domain without a DNA‐binding domain. PhcQ is an intracellular soluble regulator containing a receiver domain without a DNA‐binding site. Schell (2000) proposed a model for the QS signalling pathway of the phylotype IIA strain AW1 of *R*. *solanacearum*, which produces 3‐OH PAME as the QS signal (Flavier et al., 1997). At low levels of 3‐OH PAME, the regulator protein PhcR may interact with PhcA, leading to PhcA dysfunction. At the threshold concentration of 3‐OH PAME, the sensor histidine kinase PhcS senses 3‐OH PAME and carries out the phosphorylation by itself. This event may induce the ability of PhcS to phosphorylate the cognate PhcR, resulting in functional PhcA. Furthermore, Tang et al. (2020) demonstrated that PhcQ is involved in the dynamics of activation of PhcA in response to the bacterial density of phylotype I strain GMI1000 of *R*. *pseudosolanacearum*, which produces 3‐OH MAME as the QS signal (Kai et al., 2015). However, the effects of these regulator proteins on the transcription of QS‐dependent genes have not been validated experimentally.

In this study, we aimed to elucidate the function of PhcR and PhcQ in the regulation of QS‐dependent genes. To achieve this goal, we first created *phcR*‐deletion (Δ*phcR*) and *phcQ*‐deletion (Δ*phcQ*) mutants from the phylotype I strain OE1‐1 of *R*. *pseudosolanacearum*, which produces 3‐OH MAME as the QS signal (Kai et al., 2015), and analysed their QS‐dependent phenotypes as well as their virulence on tomato plants. We then analysed the transcriptomes of *R*. *pseudosolanacearum* strains using quantitative reverse transcription‐PCR (RT‐qPCR) and RNA‐sequencing (RNA‐seq).

## 2 RESULTS

### 2.1 Phylogenetic analysis of the deduced amino acid sequences of PhcR and PhcQ among RSSC strains

To analyse the genetic variation of PhcR and PhcQ among 34 RSSC strains (phylotype I, nine strains; phylotype IIA, six strains; phylotype IIB, five strains; phylotype III, three strains; and phylotype IV, 11 strains, including blood disease bacterial strain R229 and *R*. *syzygii* strain R24; Table S1), the deduced amino acid sequences of PhcR and PhcQ were analysed with ClustalW and phylogenetic trees were constructed with TreeView. The phylogenetic trees regarding PhcR (Figure S1a) as well as PhcQ (Figure S1b) showed that the 34 strains were divided into four clades, consistent with their phylotypes.

### 2.2 Deletion of *phcQ*, but not *phcR*, led to significant changes in QS‐dependent phenotypes

**FIGURE 1:**
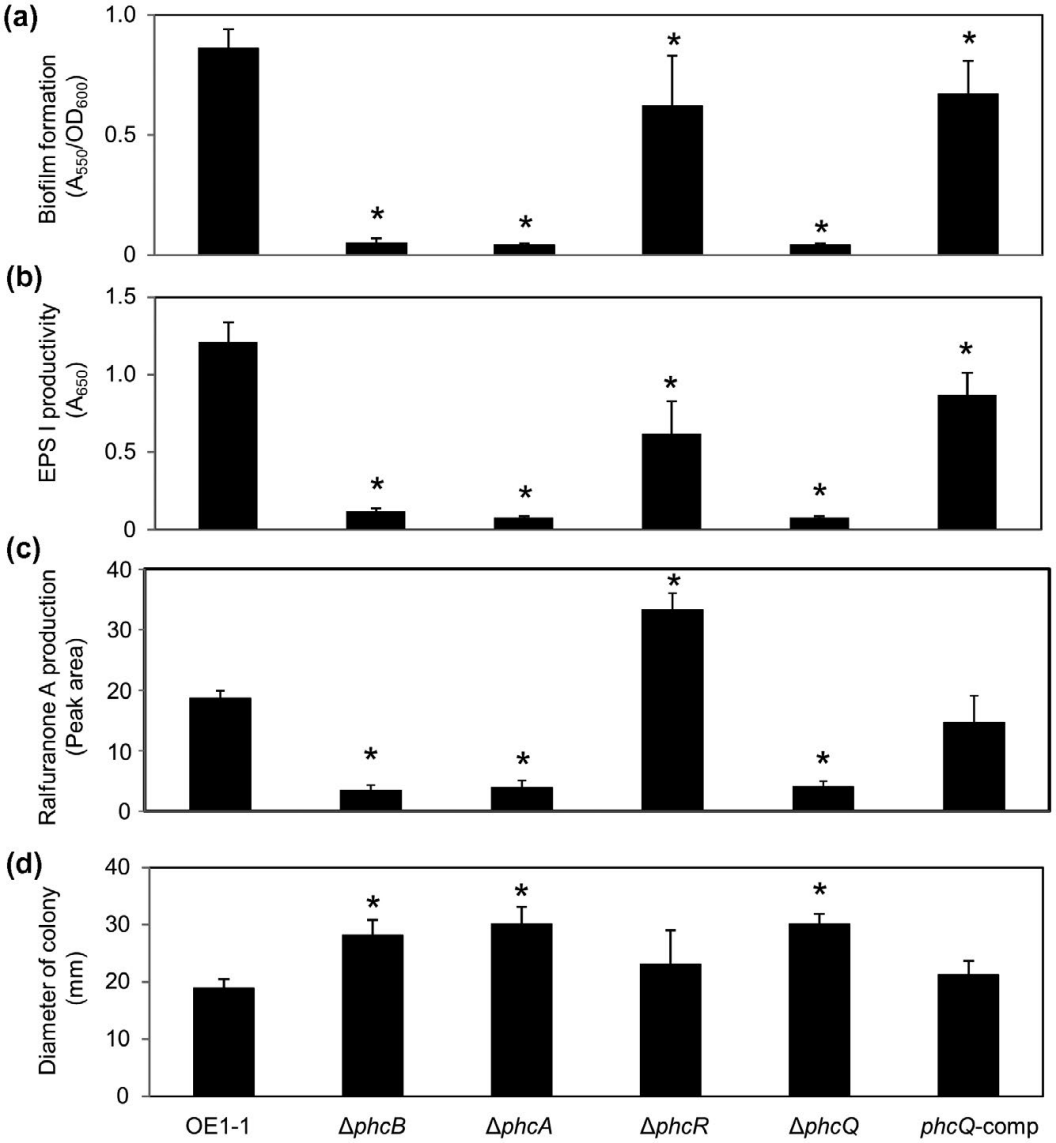
(a) Biofilm formation, (b, c) production of the major exopolysaccharide EPS I (b) and ralfuranone A (c), (d) and swimming motility of *Ralstonia pseudosolanacearum* strain OE1‐1, *phcB*‐deletion (Δ*phcB*), *phcA*‐deletion (Δ*phcA*), *phcR*‐deletion (Δ*phcR*), and *phcQ*‐deletion (Δ*phcQ*) mutants, and the Δ*phcQ* mutant transformed with native *phcQ* (*phcQ*‐comp). (a) Cells of *R. pseudosolanacearum* incubated in quarter‐strength M63 medium in wells of polyvinylchloride microtitre plates were stained with crystal violet. Three replicate experiments conducted using independent samples with seven technical replicates per experiment produced similar results. The results of a representative experiment are shown. (b) *R*. *pseudosolanacearum* strains were incubated on quarter‐strength M63 medium solidified with 0.25% agar. Three replicate experiments conducted using independent samples with five technical replicates per experiment produced similar results. The results of a representative experiment are shown. (c) *R*. *pseudosolanacearum* strains were grown for 4 days in 100 ml MGRL medium containing 3% sucrose. The results of an HPLC analysis of culture extracts are presented. The experiment was conducted three times using independently prepared samples. (d) *R*. *pseudosolanacearum* strains were grown on quarter‐strength M63 medium solidified with 0.25% agar. Three replicate experiments conducted using independent samples with five technical replicates per experiment produced similar results. The results of a representative experiment are shown. Bars indicate standard errors. Asterisks indicate values significantly different from those of OE1‐1 (*p* < .05, *t* test)

We created Δ*phcR* (Table 1) and Δ*phcQ* (Table 1) mutants of strain OE1‐1 and analysed their QS‐dependent phenotypes. We first assayed biofilm formation with crystal violet staining of *R*. *pseudosolanacearum* strains grown in quarter‐strength M63 medium. The Δ*phcR* mutant exhibited slightly less biofilm formation than wild‐type strain OE1‐1 (Figure 1a). The Δ*phcQ* mutant produced significantly less biofilm than strain OE1‐1, similar to the Δ*phcB* mutant (Table 1; Kai et al., 2015) and the Δ*phcA* mutant (Table 1; Mori et al., 2016) (*p* < .05, *t* test). The Δ*phcR* mutant produced less EPS I (Figure 1b) and more ralfuranone A (Figure 1c), which is one of the ralfuranones, than strain OE1‐1, whereas the *phcQ* deletion led to significantly reduced production of EPS I (*p* < .05, *t* test; Figure 1b) and ralfuranone A (*p* < .05, *t* test; Figure 1c), similar to the effects of *phcB* and *phcA* deletions. Compared with strain OE1‐1, the Δ*phcR* mutant, when grown on quarter‐strength M63 medium solidified with 0.25% agar, exhibited slightly enhanced swimming motility (Figure 1d). The swimming motility of the Δ*phcQ* mutant was significantly greater than that of strain OE1‐1, similar to the Δ*phcB* and Δ*phcA* mutants (*p* < .05, *t* test).

**TABLE 1:** Strains and plasmids used in this study

	Relevant characteristics	Source
Plasmids		
pUC118	Amp^r^	Takara Bio
pK18mobsacB	Km^r^, *oriT* (RP4), *sacB*, *lacZα*	Kvitko and Collmer (2011)
pUC18‐mini‐*Tn*7T‐Gm	Gm^r^	Choi et al. (2005)
pTNS2	Helper plasmid carrying T7 transposase gene	Choi et al. (2005)
pMD20delta‐phcR	pMD20 derivative carrying a 1.4‐kb DNA fragment for *phcR* deletion, Amp^r^	This study
pdelta‐phcR	pK18mobsacB derivative carrying a 1.4‐kb DNA fragment for *phcR* deletion, Km^r^	This study
pdelta‐phcQ	pK18mobsacB derivative carrying a 1.5‐kb DNA fragment for *phcR* deletion, Km^r^	This study
pUC18‐mini‐Tn*7*T‐Gm‐phcQ	pUC18‐mini‐Tn*7*T‐Gm derivative carrying a 2.8‐kb fragment for *phcR* complementation, Gm^r^	This study
*Escherichia coli* strain		
DH5α	*recA1 endA1 gyrA96 thi‐1 hsdR17supE44* Δ(*lac*)*U169*(ϕ*80lac*ΔM15)	Takara Bio
*R. solanacearum* strains		
OE1‐1	Wild‐type strain, phylotype I, race 1, biovar 4	Kanda et al. (2003)
Δ*phcB*	*phcB*‐deletion mutant of OE1‐1	Kai et al. (2015)
Δ*phcA*	*phcA*‐deletion mutant of OE1‐1	Mori et al. (2016)
Δ*phcK*	*phcK*‐deletion mutant of OE1‐1	Senuma et al. (2020)
Δ*phcR*	*phcR*‐deletion mutant of OE1‐1	This study
Δ*phcQ*	*phcQ*‐deletion mutant of OE1‐1	This study
*phcQ‐comp*	A transformant of Δ*phcQ* with pUC18‐mini‐Tn*7*T‐Gm‐phcQ containing native *phcQ*, Gm^r^	This study
*lecM*‐M	*lecM*::Tn EZ::TN <KAN‐2> mutant, Km^r^	Mori et al. (2016)

**FIGURE 2:**
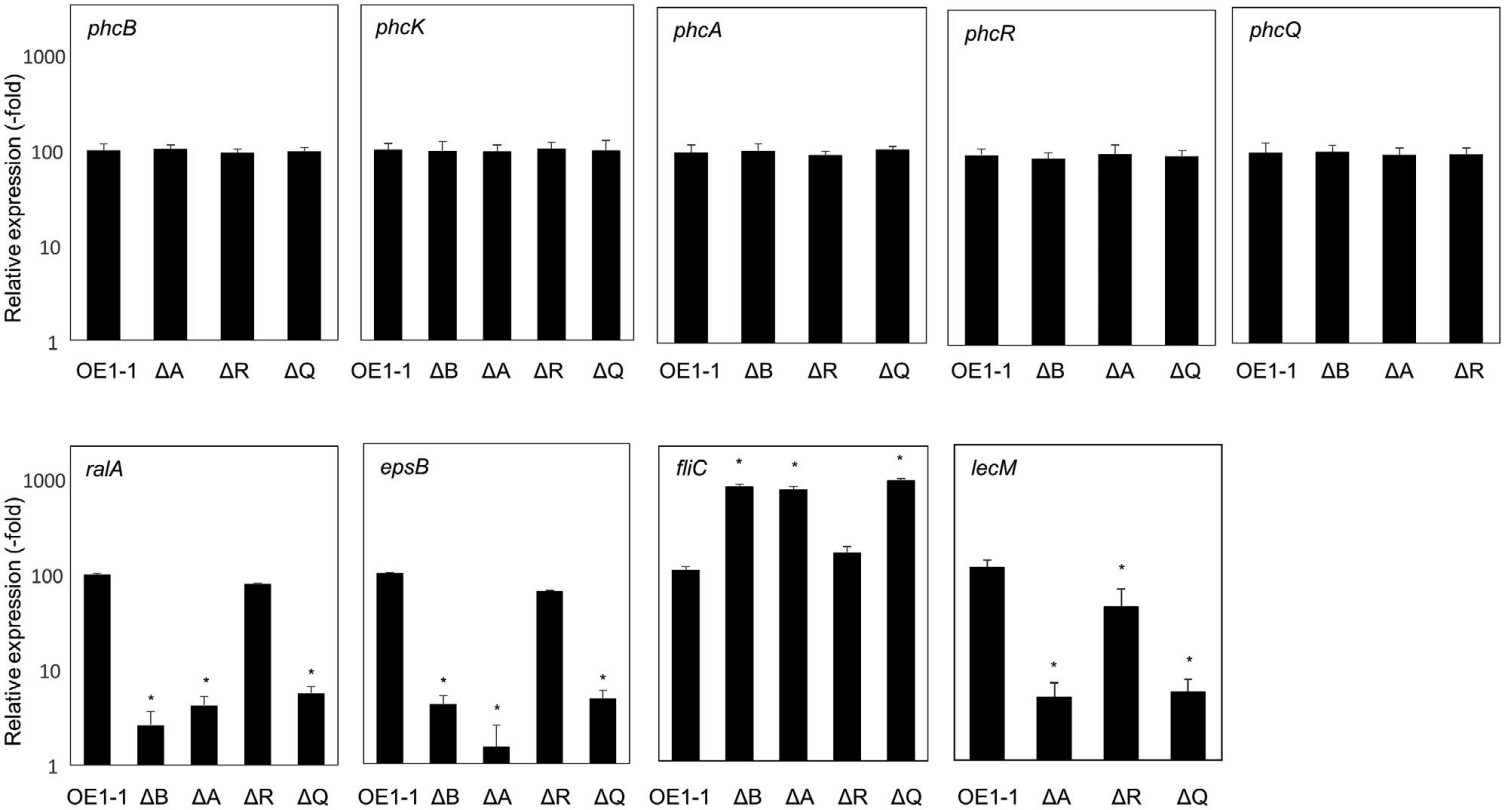
Expression levels of the quorum sensing (QS)‐related genes *phcB*, *phcK*, *phcA*, *phcR*, and *phcQ*, the positively QS‐dependent genes *ralA*, *epsB*, and *lecM*, and the negatively QS‐dependent gene *fliC* in *Ralstonia pseudosolanacearum* strain OE1‐1 and *phcA*‐deletion (ΔA), *phcR*‐deletion (ΔR), *phcQ*‐deletion (ΔQ), and *phcB*‐deletion (ΔB) mutants grown in quarter‐strength M63 medium until OD_600_ = 0.3, as determined by quantitative reverse transcription‐PCR. Two replicate experiments conducted using independent samples with eight technical replicates per experiment produced similar results. Results of a single representative sample are provided. Bars indicate standard errors. Asterisks indicate values significantly different from those of OE1‐1 (*p* < .05, *t* test)

The induced expression of two genes—*epsB*, which is part of the *eps* operon and required for EPS I biosynthesis (Huang & Schell, 1995), and *ralA*, which encodes a ralfuranone synthase (Kai et al., 2014; Wackler et al., 2011)—is dependent on QS. In addition, the expression of the flagellar motility‐related gene *fliC*, which encodes flagellin, is suppressed in the active state of QS (Tans‐Kersten et al., 2001). To analyse expression levels of these genes in *R*. *pseudosolanacearum* strains grown in quarter‐strength M63 medium until OD_600_ = 0.3, we conducted RT‐qPCR assays. Expression levels of *ralA* and *epsB* in the Δ*phcQ* mutant but not in the Δ*phcR* mutant were significantly lower than those in OE1‐1 (*p* < .05, *t* test; Figure 2). In contrast, *fliC* was more highly expressed in the Δ*phcQ* mutant, but not in the Δ*phcR* mutant, compared with the OE1‐1 strain (*p* < .05, *t* test).

### 2.3 Transformation of native PhcQ recovered QS‐dependent phenotypes of the Δ*phcQ* mutant

We next transformed the Δ*phcQ* mutant with pUC18‐mini‐Tn*7*T‐Gm‐phcQ (Table 1) harbouring the native *phcQ* gene fused with the promoter of the *phcBSRQ* operon (Kai et al., 2015) to generate the complemented Δ*phcQ* mutant strain *phcQ*‐comp (Table 1). Transformation of the Δ*phcQ* mutant with the pUC18‐mini‐Tn*7*T‐Gm‐phcQ construct led to enhanced biofilm formation (Figure 1a) and production of EPS I (Figure 1b) and ralfuranone A (Figure 1c) and reduced swimming motility (Figure 1d).

### 2.4 Deletion of *phcQ*, but not *phcR*, led to a loss of bacterial virulence

**FIGURE 3:**
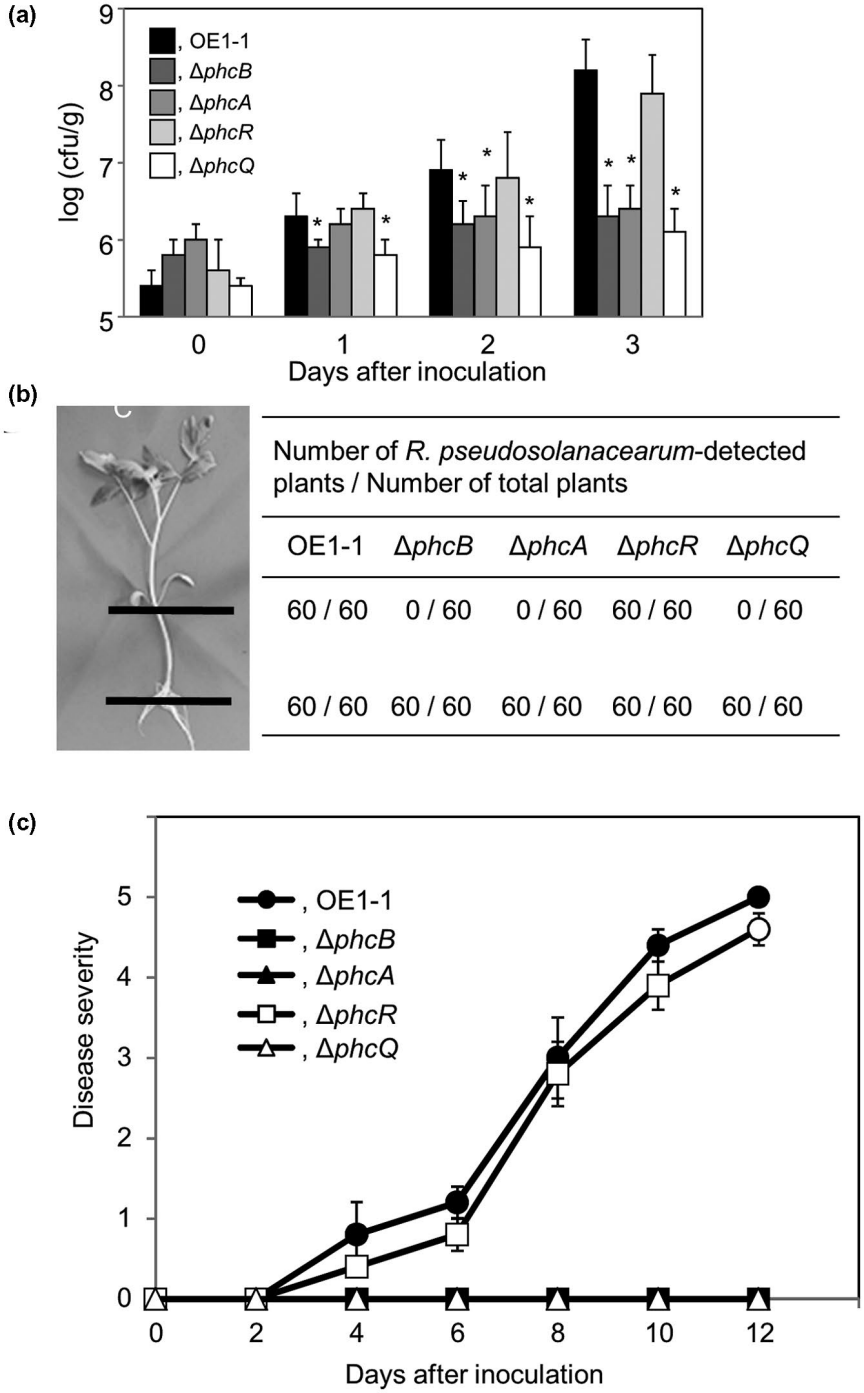
Population, behaviour, and virulence of *Ralstonia pseudosolanacearum* strains OE1‐1, Δ*phcB*, Δ*phcA*, Δ*phcR*, and Δ*phcQ* in 8‐week‐old tomato plants inoculated by the root‐dip method. (a) Populations of *R. pseudosolanacearum* strains in tomato roots. Data are presented as the mean ±*SD* of five trials. Asterisks indicate values significantly different from those of wild‐type strain OE1‐1. cfu, colony‐forming unit. (b) Behaviour of *R. pseudosolanacearum* strains in tomato roots and stems 10 days after root‐dip inoculation as determined by a plate‐printing assay (Hayashi et al., 2019). (c) Virulence of *R. pseudosolanacearum* strains. Plants were rated according to the following disease index scale: 0, no wilting; 1, 1%–25% wilting; 2, 26%–50% wilting; 3, 51%–75% wilting; 4, 76%–99% wilting; and 5, dead. For each bacterial strain, three replicate experiments conducted using independent samples with 12 technical replicates per experiment produced similar results. Results of a single representative sample are provided. Data are presented as the mean ±*SD* of 12 replicates

To investigate the effects of *phcR* and *phcQ* on the virulence of strain OE1‐1, we inoculated 5‐week‐old tomato plants with *R*. *pseudosolanacearum* strains by the root‐dip method and then assayed the population dynamics and behaviour of these strains in the tomato plants as well as disease development. The population of the Δ*phcQ* mutant at 3 days after inoculation (DAI) was significantly smaller than that of OE1‐1 and the Δ*phcR* mutant, similar to Δ*phcB* and Δ*phcA* mutants (*p* < .05, *t* test; Figure 3a). In a plate‐printing assay, we detected OE1‐1 and the Δ*phcR* mutant in the inoculated roots and stems of tomato plants, whereas no Δ*phcQ* was observed beyond the inoculated roots, similar to Δ*phcB* and Δ*phcA* mutants (Figure 3b). The tomato plants inoculated with the OE1‐1 strain exhibited wilt symptoms at 5 DAI and died by 10 DAI (Figure 3c). The Δ*phcQ* mutant was not virulent on tomato plants 10 DAI, similar to the Δ*phcB* and Δ*phcA* mutants, whereas the virulence of the Δ*phcR* mutant resembled that of the OE1‐1 strain.

### 2.5 Deletion of *phcR* or *phcQ* did not influence the regulation of QS‐related genes

The Δ*phcQ* mutant exhibited significantly changed QS‐dependent phenotypes including the virulence compared to strain OE1‐1, though the QS‐dependent phenotypes of the Δ*phcR* mutant were largely unchanged (Figures 2 and 3). To analyse the effects of deletion of *phcR* and *phcQ* on the gene expression of *phcB*, *phcK*, and *phcA* (QS‐related genes), we monitored the expression levels of QS‐related genes in *R*. *pseudosolanacearum* strains grown in quarter‐strength M63 medium until OD_600_ = 0.3 by RT‐qPCR. No significant differences in expression levels of *phcB*, *phcK*, and *phcA* were detected among the Δ*phcQ* mutant, the Δ*phcR* mutant, and OE1‐1 (*p* < .05, *t* test; Figure 2). Furthermore, expression levels of *phcQ* and *phcR* in the Δ*phcR* mutant and Δ*phcQ* mutant, respectively, were similar to those in OE1‐1 (*p* < .05, *t* test).

### 2.6 RNA‐seq transcriptome analysis of *R. pseudosolanacearum* strains

The deletion of *phcR* or *phcQ* did not influence the regulation of QS‐related genes (Figure 2). To analyse the effects of *phcR* and *phcQ* deletion on the regulation of QS‐dependent genes, we performed an RNA‐seq transcriptome analysis of *R*. *pseudosolanacearum* strains grown in quarter‐strength M63 medium until OD_600_ = 0.3. Mapping of RNA‐seq reads of the OE1‐1 strain to the GMI1000 genome (Salanoubat et al., 2002) resulted in the identification of 4,437 protein‐coding transcripts (Table S2). To extract genes with significant expression changes, the following thresholds were applied: *q* < .05 and |log(fold change [FC])| ≥ 2. Compared with their expression levels in OE1‐1, 371 and 174 genes in the Δ*phcB* mutant were significantly down‐regulated and up‐regulated, respectively (Figure S2). Among them, 345 and 163 genes in the Δ*phcA* mutant were significantly down‐regulated and up‐regulated, respectively, and were thus inferred to be positively and negatively QS‐dependent genes, respectively.

**FIGURE 4:**
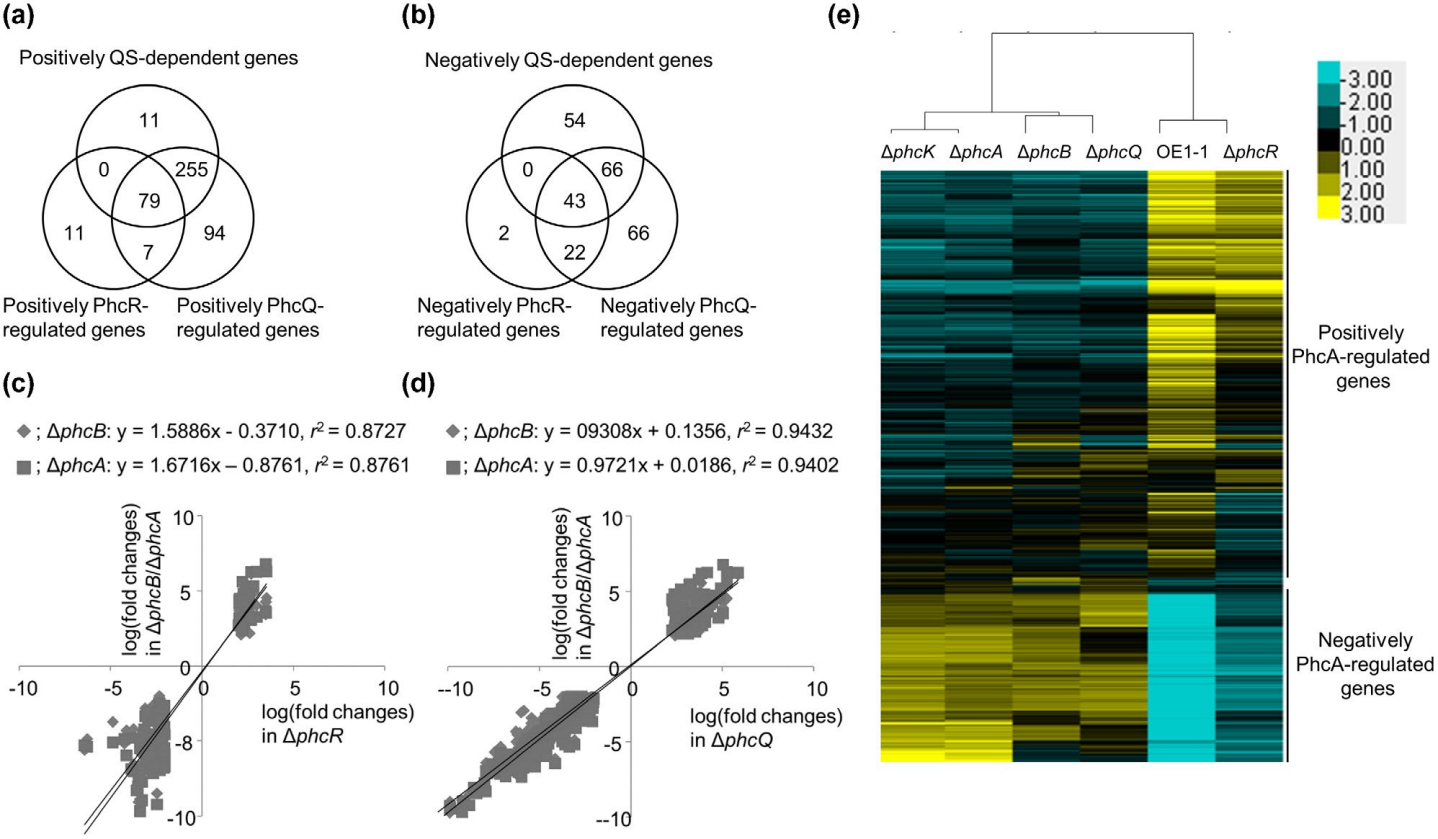
RNA‐sequencing transcriptome analysis of quorum sensing (QS)‐dependent genes of *Ralstonia pseudosolanacearum* strains grown in quarter‐strength M63 medium until OD_600_ = 0.3. (a) The number of genes exhibiting log(fold change) ≤ −2 in the *phcB*‐deletion (Δ*phcR*) and *phcQ*‐deletion (Δ*phcQ*) mutants relative to their expression levels in strain OE1‐1 (*q* < .05). (b) The number of genes exhibiting log(fold change) ≥ 2 in the Δ*phcR* and Δ*phcQ* mutants relative to their expression levels in strain OE1‐1 (*q* < .05). (c) Correlation of expression levels of QS‐dependent genes between *R. pseudosolanacearum* mutants: Δ*phcR* versus Δ*phcB* or Δ*phcA* mutants. (d) Correlation of expression levels of QS‐dependent genes between *R. pseudosolanacearum* mutants: Δ*phcQ* versus Δ*phcB* or Δ*phcA*. (e) Hierarchical clustering of relative expression levels of PhcA‐regulated genes in *R. pseudosolanacearum* strains OE1‐1, Δ*phcB*, Δ*phcK*, Δ*phcA*, Δ*phcR*, and Δ*phcQ*. Fragments per kilobase of exon per million fragments mapped values from *R. pseudosolanacearum* strains OE1‐1, Δ*phcB*, Δ*phcK*, Δ*phcA,* Δ*phcR*, and Δ*phcQ* were normalized prior to analysis of differentially expressed genes

The transcriptome analysis with RNA‐seq showed that 97 genes in the Δ*phcR* mutant were significantly down‐regulated compared with their expression levels in OE1‐1, and were thus inferred to be positively PhcR‐regulated genes (Figure 4a; Table S3), while 67 genes (negatively PhcR‐regulated genes) in the Δ*phcR* mutant were significantly up‐regulated (Figure 4b; Table S3). Among the positively PhcR‐regulated genes, 79 genes, including *norB*, *lecM*, and *xpsR*, were positively QS‐dependent genes (Figure 4a; Table S3). Among the negatively PhcR‐regulated genes, 43 genes, including some flagellin biosynthesis‐related genes and chemotaxis‐related genes, were negatively QS‐dependent genes (Figure 4b; Table S3). The log(FC) values of these PhcR‐regulated QS‐dependent genes between strain OE1‐1 and the Δ*phcR* mutant were moderately correlated with those between OE1‐1 and the Δ*phcB* mutant (*y*, log(FC) of Δ*phcB*; *x*, log(FC) of Δ*phcR*; *y* = 1.5886*x* − 0.371, *r* ^2^ = .8727; Figure 4c) or the Δ*phcA* mutant (*y*, log(FC) of Δ*phcA*; *x*, log(FC) of Δ*phcR*; *y* = 1.6716*x* − 0.4498, *r* ^2^ = .8761; Figure 4c). It is thus thought that PhcR is partially involved in the regulation of 79 (22.9%) and 43 (26.4%) of the positively and negatively QS‐dependent genes, respectively.

The transcriptome analysis with RNA‐seq showed that in the Δ*phcQ* mutant, 435 positively PhcQ‐regulated genes were significantly down‐regulated (Figure 4a; Table S4) and 197 negatively PhcQ‐regulated genes were significantly up‐regulated relative to strain OE1‐1 (Figure 4b; Table S4). Among the positively PhcQ‐regulated genes, 334 genes were positively QS‐dependent genes (Figure 4a; Table S4). Among the negatively PhcQ‐regulated genes, 109 genes were negatively QS‐dependent genes (Figure 4b; Table S4). A strong positive correlation of the expression of these QS‐dependent genes among PhcQ‐regulated genes was observed between the Δ*phcQ* mutant and the Δ*phcB* mutant (*y*, log(FC) of Δ*phcB*; *x*, log(FC) of Δ*phcQ*; *y* = 0.9308*x* + 0.1356, *r* ^2^ = .9432; Figure 4d) and between the Δ*phcQ* mutant and the Δ*phcA* mutant (*y*, log(FC) of Δ*phcA*; *x*, log(FC) of Δ*phcQ*; *y* = 0.9721*x* + 0.0186, *r* ^2^ = .9402; Figure 4d). Therefore, PhcQ is mainly involved in the regulation of 334 (96.8%) and 109 (66.9%) of the positively and negatively QS‐dependent genes, respectively.

We then carried out hierarchical clustering of the QS‐related gene‐deletion mutants and the Δ*phcR* and Δ*phcQ* mutants based on their relative expression levels normalized against those of QS‐dependent genes. In the resulting dendrogram, the Δ*phcR* mutant clustered with strain OE1‐1, whereas the Δ*phcQ* mutant grouped with the Δ*phcB* mutant, and Δ*phcA* clustered separately with the *phcK*‐deletion (Δ*phcK*) mutant (Figure 4e).

### 2.7 Deletion of *phcQ* led to a significant reduction in 3‐OH MAME content

**FIGURE 5:**
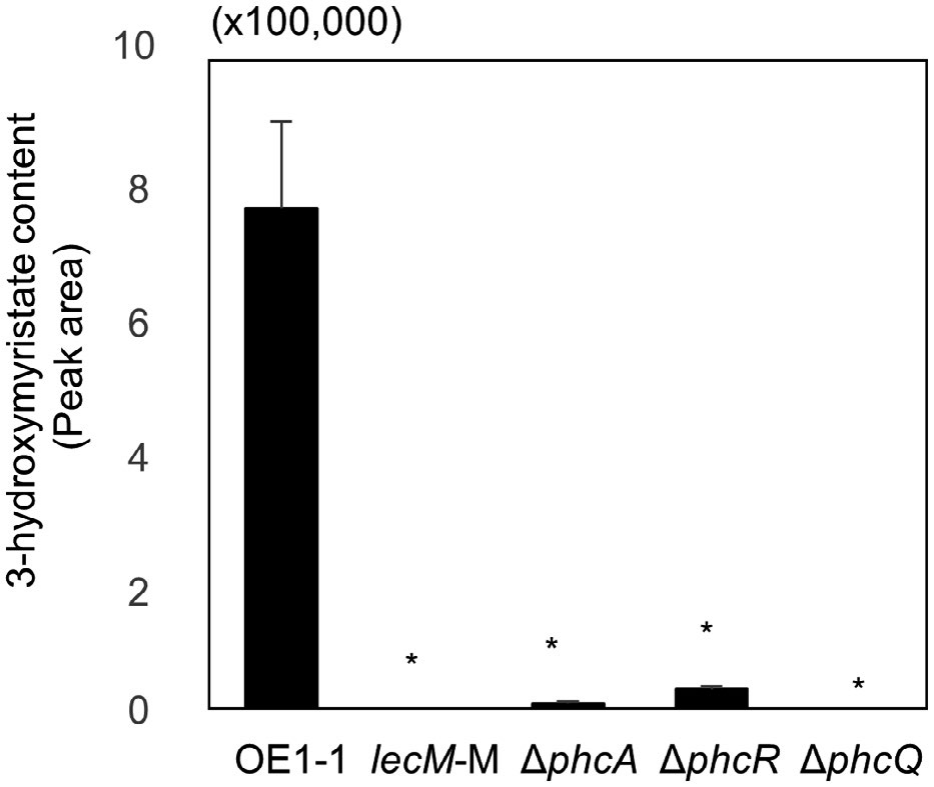
Content of methyl 3‐hydroxymyristate (3‐OH MAME) purified from *Ralstonia pseudosolanacearum* strains OE1‐1, Δ*phcR*, Δ*phcQ*, Δ*phcA*, and *lecM‐*M grown on BG agar plates for 24 hr at 30 °C. Data are presented as the mean ±*SD* of four trials. Asterisks indicate values significantly different from those of OE1‐1 (*p* < .05, *t* test)

Tang et al. (2020) demonstrated that PhcQ contributes to the synthesis of 3‐OH MAME by strain GMI1000. In the active state of QS, expression of *lecM*, which encodes the lectin LecM, is induced and LecM affects the activation of QS through regulating the stability of secreted 3‐OH MAME (Hayashi et al., 2019a). Deletion of *phcR* or *phcQ* did not influence the regulation of QS‐related genes including *phcB* (Figure 2). We assayed 3‐OH MAME content purified from *R*. *pseudosolanacearum* strains. The *lecM*‐M mutant exhibited a significantly lower 3‐OH MAME content than strain OE1‐1, similar to the Δ*phcQ* mutant (*p* < .05, Figure 5). Though the Δ*phcR* mutant exhibited significantly lower 3‐OH MAME content compared to strain OE1‐1, 3‐OH MAME content in the Δ*phcR* mutant was higher than that in other mutants, including the Δ*phcA* mutant.

To analyse the expression levels of *lecM* in *R*. *pseudosolanacearum* strains grown in quarter‐strength M63 medium until OD_600_ = 0.3, we conducted RT‐qPCR assays. The expression levels of *lecM* in the Δ*phcQ* and Δ*phcA* mutants was significantly lower than in strain OE1‐1 (*p* < .05, *t* test; Figure 2). Though the expression level of *lecM* in the Δ*phcR* mutant was significantly lower than in strain OE1‐1, the *lecM* expression level in the Δ*phcR* mutant was higher than in the Δ*phcA* mutant.

### 2.8 Deletion of *phcR* led to a slight change in expression levels of QS‐dependent genes at lower bacterial density

**FIGURE 6:**
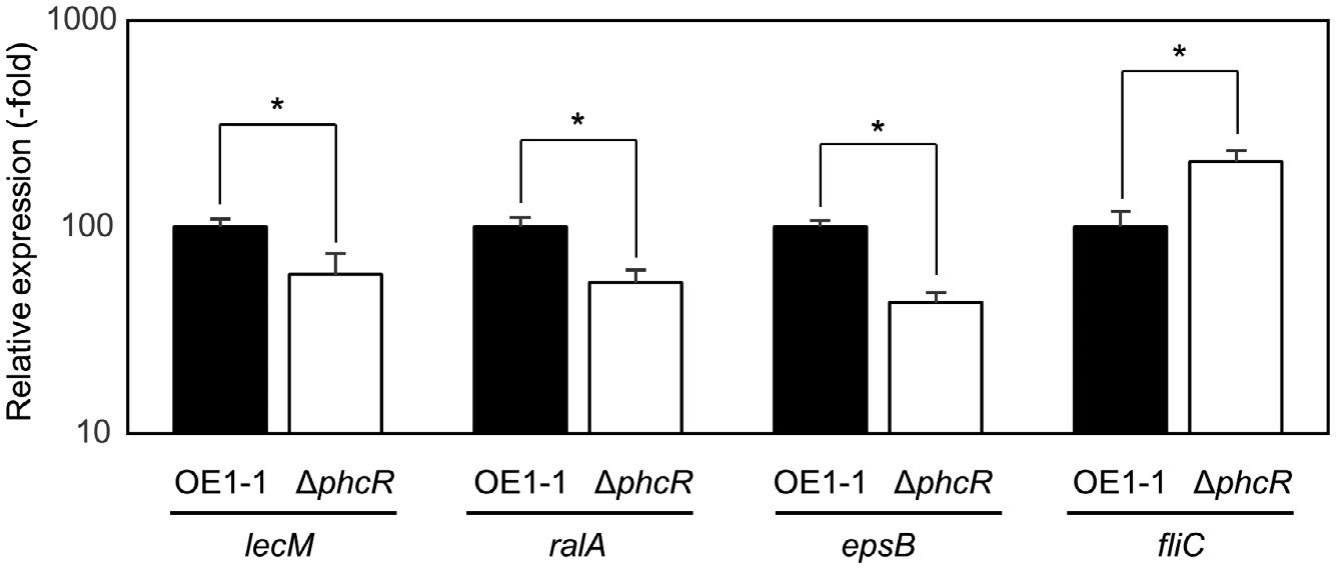
Expression of the positively QS‐dependent genes *lecM, ralA*, and *epsB*, and the negatively QS‐dependent gene *fliC* in *Ralstonia pseudosolanacearum* strains OE1‐1 and Δ*phcR* grown in quarter‐strength M63 medium until OD_600_ = 0.01, as determined by quantitative reverse transcription‐PCR. Two replicate experiments conducted using independent samples with eight technical replicates per experiment produced similar results. Results of a single representative sample are provided. Bars indicate standard errors. Asterisks indicate values significantly different from those of OE1‐1 (*p* < .05, *t* test)

In the inactive state of QS, PhcR reportedly inhibits PhcA function in strain AW1 (Schell, 2000). To analyse the influence of *phcR* deletion on expression levels of QS‐dependent genes in the inactive state of QS, using RNA isolated from the Δ*phcR* mutant and strain OE1‐1 grown in quarter‐strength M63 medium until OD_600_ = 0.01, we conducted RT‐qPCR assays to assess relative expression levels of *lecM*, *ralA*, *epsB*, and *fliC* in the Δ*phcR* mutant compared to strain OE1‐1. The expression levels of the positively QS‐dependent genes *lecM*, *ralA*, and *epsB* were slightly but significantly lower in the Δ*phcR* mutant than in strain OE1‐1 (*p* < .05, *t* test; Figure 6). In addition, the expression level of the negatively QS‐dependent *fliC* was significantly higher in the Δ*phcR* mutant than in strain OE1‐1 (*p* < .05, *t* test).

### 2.9 Exogenous 3‐OH MAME application did not lead to a change in the QS‐dependent phenotypes of Δ*phcR* and Δ*phcQ* mutants

**FIGURE 7:**
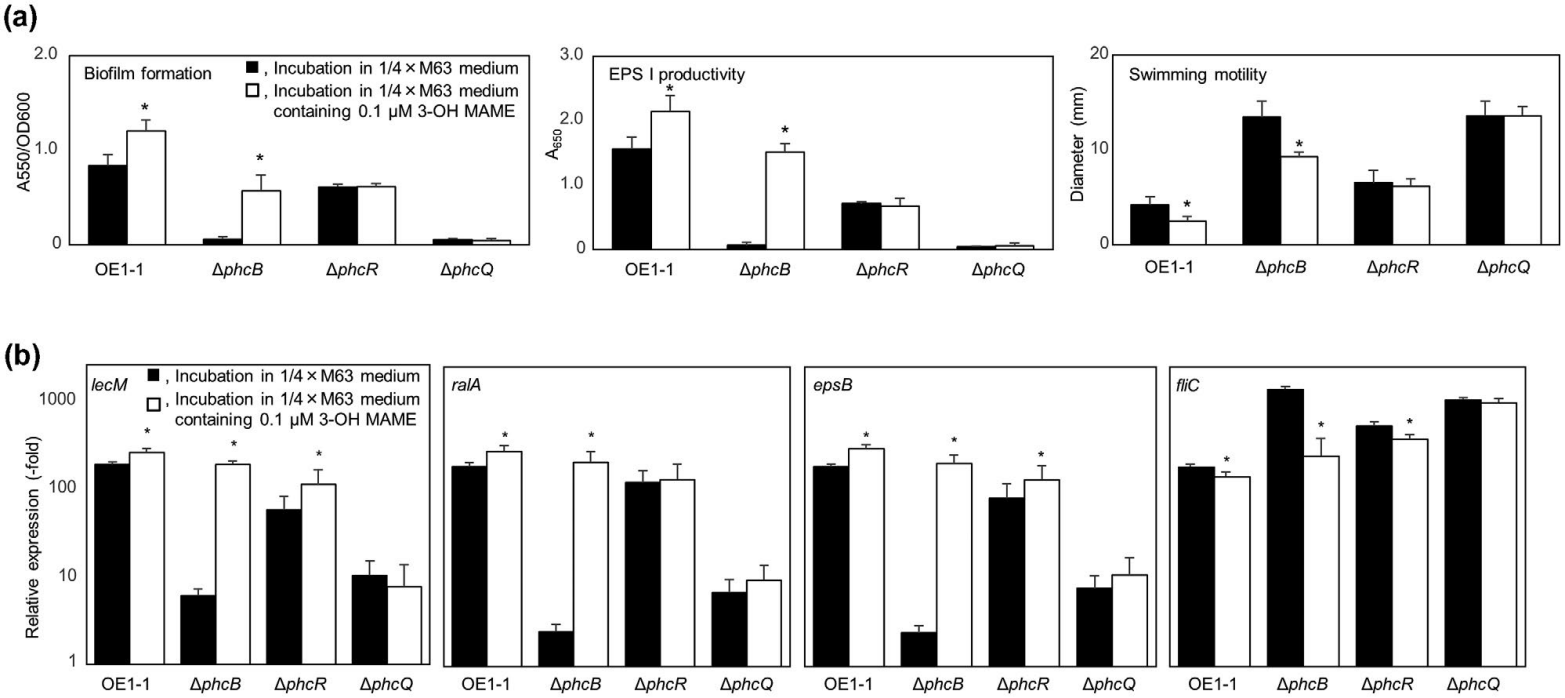
(a) Influence of extracellular methyl 3‐hydroxymyristate (3‐OH MAME) application on quorum sensing (QS)‐dependent phenotypes, biofilm formation, exopolysaccharide EPS I production, and swimming motility of *Ralstonia pseudosolanacearum* strains OE1‐1, Δ*phcB*, Δ*phcR*, and Δ*phcQ*. (b) Expression levels of the QS‐dependent genes *epsB*, *ralA*, and *fliC* in the same strains. (a) For the biofilm formation and EPS I production assays, *R. pseudosolanacearum* strains were grown in quarter‐strength M63 or quarter‐strength M63 medium containing 0.1 µM 3‐OH MAME. For the swimming motility assay, 5‐μl aliquots of cell suspensions at 5 × 10^5^ cfu/ml were added to the centre of plates containing quarter‐strength M63 medium solidified with 0.25% agar, and the swimming‐area diameters were measured at 24 hr postinoculation. Three replicate experiments conducted using independent samples with seven technical replicates per biofilm formation experiment and five technical replicates per EPS I production or swimming motility experiment produced similar results. The results of a representative experiment are shown. Bars indicate standard errors. Asterisks indicate values significantly different from those of controls without 3‐OH MAME (*p* < .05, *t* test). (b) Total RNA was extracted from bacterial cells grown until OD_600_ = 0.01. Bars indicate standard errors. Asterisks indicate values significantly different from those of OE1‐1 (*p* < .05, *t* test)

QS activity in strain OE1‐1 is dependent on the exogenous levels of 3‐OH MAME (Hayashi et al., 2019b; Kai et al., 2015). We examined the influence of exogenous 3‐OH MAME application on QS‐dependent phenotypes, biofilm formation, EPS I production, and swimming motility of these mutants. Exogenous application of 3‐OH MAME at a concentration of 0.1 μM enhanced biofilm formation and EPS I production and reduced swimming motility of strain OE1‐1 and the Δ*phcB* mutant (*p* < .05, *t* test; Figure 7a). However, exogenous 3‐OH MAME application had no effect on these QS‐dependent phenotypes of Δ*phcR* and Δ*phcQ* mutants.

To analyse the influence of exogenous 3‐OH MAME application on the expression levels of the QS‐dependent genes *lecM*, *ralA*, *epsB*, and *fliC*, we conducted RT‐qPCR assays using *R*. *pseudosolanacearum* strains grown in quarter‐strength M63 medium containing 3‐OH MAME at a concentration of 0.1 μM until OD_600_ = 0.3. Exogenous 3‐OH MAME application significantly enhanced the expression levels of *lecM*, *ralA*, and *epsB* and significantly reduced the expression levels of *fliC* in strain OE1‐1 and the Δ*phcB* mutant but not in the Δ*phcQ* mutant (*p* < .05, *t* test; Figure 7b). In contrast, in the Δ*phcR* mutant, exogenous 3‐OH MAME application slightly but significantly enhanced the expression levels of *lecM* and *epsB*, but not *ralA*, and slightly but significantly reduced the expression levels of *fliC* (*p* < .05, *t* test).

### 2.10 PhcA was involved in the negative control of siderophore‐mediated iron acquisition activity, independently of PhcR and PhcQ

**FIGURE 8:**
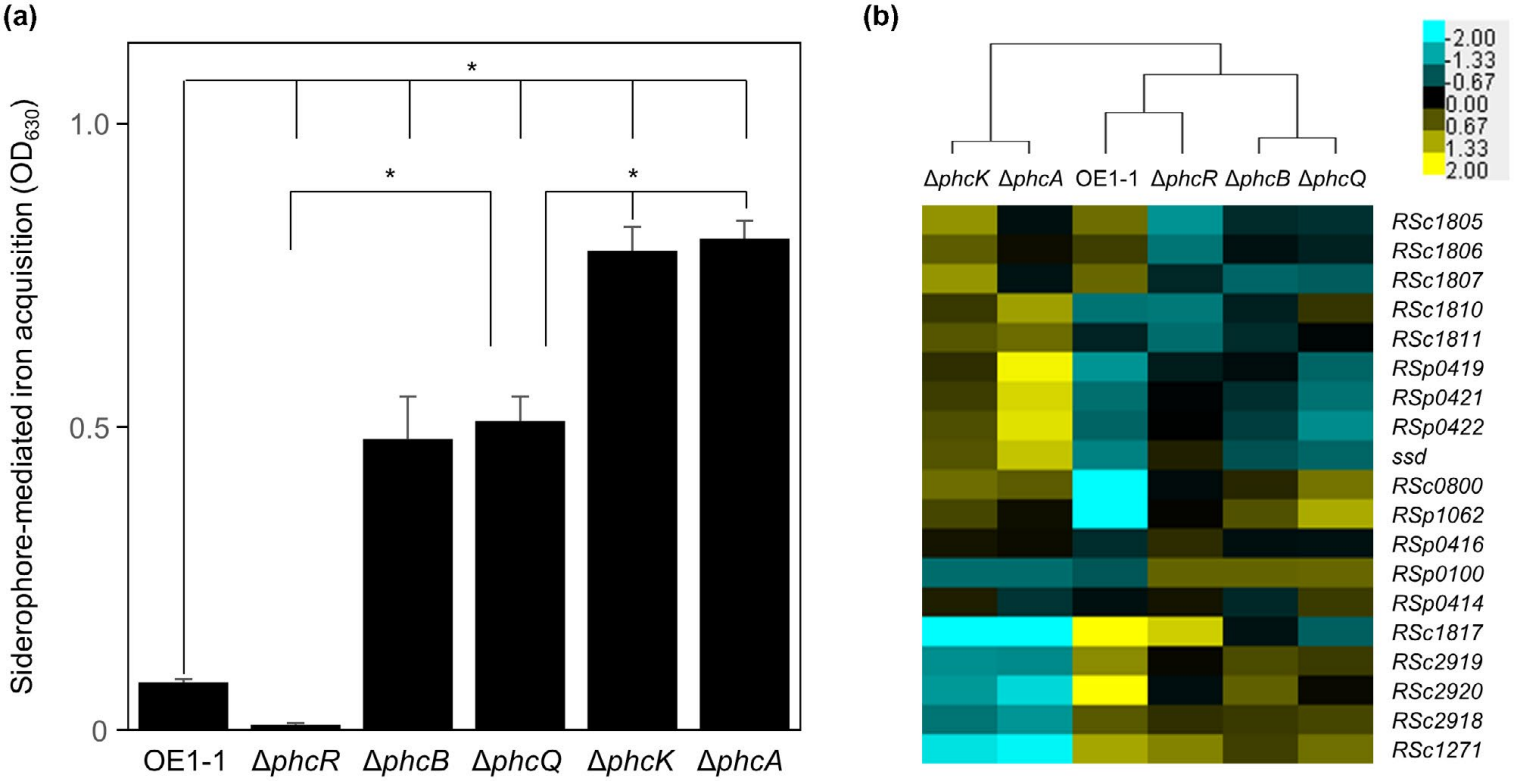
(a) Siderophore‐mediated iron acquisition activity and (b) hierarchical clustering of relative expression levels of genes involved in siderophore‐mediated iron acquisition in *Ralstonia pseudosolanacearum* strains OE1‐1, Δ*phcB*, Δ*phcK*, Δ*phcA*, Δ*phcR*, and Δ*phcQ* grown in quarter‐strength M63 medium until OD_600_ = 0.3. (a) The siderophore‐mediated iron acquisition activity was calculated by subtracting the absorbance value of the reference from the total absorbance at 630 nm (A_630_). Three replicate experiments conducted using independent samples with eight technical replicates per experiment produced similar results. Results of a single representative sample are provided. Asterisks indicate values significantly different between strains (*p* < .05, *t* test). (b) Fragments per kilobase of exon per million fragments mapped values from *R. pseudosolanacearum* strains OE1‐1, Δ*phcB*, Δ*phcK*, Δ*phcA,* Δ*phcR*, and Δ*phcQ* based on our RNA‐sequencing transcriptome analysis were normalized prior to analysis of differentially expressed genes. The average value of three replicates per strain was used

RSSC strains produce micacocidin and staphyloferrin B as siderophores to acquire iron from extracellular environments (Bhatt & Denny, 2004; Kreutzer et al., 2011). The RSp0424 gene (*ssd*), which encodes diaminopimelate decarboxylase, required for staphyloferrin B production, is negatively regulated by PhcA (Bhatt & Denny, 2004). To analyse the influence of PhcR and PhcQ on siderophore‐mediated iron acquisition activity, we measured the siderophore‐mediated iron acquisition activity of *R*. *pseudosolanacearum* strains. The Δ*phcB* and Δ*phcQ* mutants had significantly higher siderophore‐mediated iron acquisition activity than wild‐type strain OE1‐1 (*p* < .05, *t* test; Figure 8a). The deletion of *phcK* or *phcA* significantly enhanced siderophore‐mediated iron acquisition activity to a greater extent than did the deletion of *phcB* or *phcQ* (*p* < .05, *t* test). On the other hand, the Δ*phcR* mutant had significantly lower siderophore‐mediated iron acquisition activity than wild‐type strain OE1‐1.

We carried out hierarchical clustering of QS‐related gene‐deletion mutants based on their relative expression levels normalized against those of siderophore‐mediated iron acquisition activity‐related genes (Table S5). The expression patterns of genes RSc1271 (*RSc1271*), RSc2918 (*RSc2918*), RSc2919 (*RSc2919*), RSc2920 (*RSc2920*), RSp0419 (*RSp0419*), RSc0421 (*RSc0421*), and RSp0422 (*RSp0422*) as well as *ssd* in the Δ*phcK* and Δ*phcA* mutants were different from those in strain OE1‐1 and other mutants (Figure 8b). In the resulting dendrogram, the Δ*phcR* mutant clustered with strain OE1‐1, whereas the Δ*phcQ* mutant grouped with the Δ*phcB* mutant, and Δ*phcA* clustered separately with the *phcK* mutant. However, the expression patterns of genes RSc1805 (*RSc1805*), RSc1806 (*RSc1806*), and RSp0100 (*RSp0100*) in the Δ*phcR* mutant were different from those in strain OE1‐1.

## 3 DISCUSSION

Balancing selection is a type of positive selection that favours the maintenance of a high genetic diversity within a given population and functions through spatial/temporal heterogeneity as well as overdominant selection and frequency‐dependent selection (Charlesworth, 2006; Hedrick, 2012; Stoeckel et al., 2012). Under balancing selection, the *phcBSRQ* operon plays a significant role in virulence of RSSC strains. (Castillo & Agathos, 2019). PhcS is subject to strong selection from the plant host (Guidot et al., 2014). Based on the deduced PhcB and PhcS amino acid sequences, RSSC strains are divided into two groups according to their QS signal types (Kai et al., 2015). In contrast, the phylogenetic analysis based on the deduced amino acid sequences of PhcR and PhcQ indicates that RSSC strains can be divided according to their phylotypes, similar to that of PhcA (Senuma et al., 2020). Overall, it is thought that extracellular communications through the production and sensing of QS signals reveal the unique evolution among RSSC strains independently of their phylotypes. The following intracellular signalling is under balancing selection according to their phylotypes and is involved in bacterial environmental fitness including virulence.

PhcQ is reportedly involved in the dynamics of activation of PhcA in the active state of QS (Tang et al., 2020). PhcQ is required for systemic infectivity of strain OE1‐1 in tomato plants and its virulence on tomato plants, similar to the other QS cascade regulatory elements. The present transcriptome analysis results of the Δ*phcQ* mutant imply that PhcQ is involved in the regulation of 96.8% and 66.9% of positively and negatively QS‐dependent genes, respectively. Furthermore, the expression levels of these PhcQ‐regulated genes in the Δ*phcQ* mutant were strongly correlated with those in Δ*phcB* or Δ*phcA*. We previously demonstrated that the putative sensor histidine kinase PhcK is required for full *phcA* expression independently of 3‐OH MAME content (Senuma et al., 2020). However, the deletion of *phcQ* had no effect on the expression level of *phcA*. In the hierarchical cluster dendrograms of PhcA‐regulated genes in QS‐related gene‐deletion mutants, the Δ*phcQ* mutant clustered with the *phcB* mutant, and the Δ*phcA* mutant grouped with the Δ*phcK* mutant. Furthermore, the exogenous application of 3‐OH MAME did not lead to a change in the expression levels of QS‐dependent genes and QS‐dependent phenotypes of the Δ*phcQ* mutant. It is thus thought that PhcQ may mainly contribute to the regulation of 96.8% and 66.9% of positively and negatively QS‐dependent genes, respectively, by PhcA, dependent on 3‐OH MAME content.

The supernatant of the *phcQ* mutant generated from strain GMI1000 does not induce the expression of the positively QS‐dependent *xpsR*, demonstrating involvement of PhcQ in 3‐OH MAME production (Tang et al., 2020). However, the influence of extracellular 3‐OH MAME application on the expression levels of QS‐related genes and other QS‐dependent genes in the Δ*phcQ* mutant is not experimentally validated. In the present study, PhcQ was involved in the induced expression of *lecM* but not *phcB*, thereby influencing the stability of secreted 3‐OH MAME and thus PhcA activation by QS. Furthermore, *phcQ* deletion significantly reduced QS‐inducible production of secondary metabolites, EPS I and ralfuranones, which are associated with the feedback loop of QS‐dependent gene regulation (Hayashi et al., 2019b; Mori et al., 2018). It is thus thought that *phcQ* deletion leads to a change in the regulation of QS‐dependent genes through extracellular 3‐OH MAME content and the feedback loop through EPS I and ralfuranones as well as PhcA activation.

The present transcriptome analysis results showed that PhcR is involved in the regulation of 22.9% and 26.4% of positively and negatively QS‐dependent genes, respectively, in strain OE1‐1. However, the expression levels of these genes in the Δ*phcR* mutant were moderately correlated with those in Δ*phcB* or Δ*phcA*. The exogenous application of 3‐OH MAME did not lead to a significant change in the QS‐dependent phenotypes of Δ*phcR*, but led to a slight but significant change in the expression levels of the QS‐dependent genes *lecM*, *epsB*, and *fliC*, but not *ralA*, in the Δ*phcR* mutant. Our transcriptome analysis showed that *phcR* deletion led to a slight but significant change in the expression levels of *lecM*, *epsB*, and *fliC*, but not *ralA*. As PhcQ mainly contributes to the regulation of these QS‐dependent genes, dependent on 3‐OH MAME content, the change in expression levels of *lecM*, *epsB*, and *fliC* in the Δ*phcR* mutant upon 3‐OH MAME treatment may result from enhanced function of PhcQ in the regulation of QS‐dependent genes. It is thus thought that PhcR may partially contribute to the regulation of 22.9% and 26.4% of positively and negatively QS‐dependent genes, respectively, by PhcA, dependently on 3‐OH MAME content. This leads to partial influences on QS‐dependent phenotypes of the Δ*phcR* mutant but not its systemic infectivity and virulence on tomato plants.

The QS model of strain AW1 proposed by Schell (2000) suggests that in the inactive state of QS, the expression of positively and negatively QS‐dependent genes should be higher and lower, respectively, in the Δ*phcR* mutant than in the wild type. However, regardless of bacterial density, the Δ*phcR* mutant exhibited slightly lower and higher expression levels of positively and negatively QS‐dependent genes, respectively, compared to the wild‐type strain OE1‐1 (Figures 2 and 6), suggesting PhcR partially contributes to the regulation of QS‐dependent genes by PhcA. Clough et al. (1997) demonstrated that the deletion of *phcR* has no obvious effect on the production of EPS I by strain AW1 incubated in EG medium under shaking. The phylogenetic analysis using the deduced amino acid sequences of PhcR indicated that PhcR‐mediated intracellular signalling pathways involved in bacterial environmental fitness may be under balancing selection according to phylotypes of RSSC strains. Incubation under different conditions may thus lead to differences in PhcR function in QS‐dependent phenotypes between strains OE1‐1 (phylotype I) and AW1 (phylotype IIA), though further experimental validations are required.

Strain AW1 produces staphyloferrin B as a siderophore, and *ssd*, which is negatively regulated by PhcA, is required for staphyloferrin B production and siderophore‐mediated iron acquisition activity (Bhatt & Denny, 2004). The strains used in the present study showed siderophore‐mediated iron acquisition activity in the order Δ*phcA* ≈ Δ*phcK* < Δ*phcB* ≈ Δ*phcQ* < OE1‐1 < Δ*phcR*. PhcA regulated not only *ssd* but also *RSc1271*, *RSc2918*, *RSc2919*, *RSc2920*, *RSp0419*, and *RSc0421*, independently of PhcR and PhcQ. In the hierarchical cluster, the Δ*phcA* mutant grouped with the Δ*phcK* mutant, and Δ*phcQ* clustered separately with the *phcB* mutant. Furthermore, though the Δ*phcR* mutant also clustered with strain OE1‐1 instead of with the other QS‐deficient mutants in the hierarchical cluster, the expression patterns of *RSc1805, RSc1806*, and *RSp0100* in the Δ*phcR* mutant were different from those in strain OE1‐1. PhcR, PhcQ, and PhcA were thus all found to be independently involved in the control of siderophore‐mediated iron acquisition activity. Furthermore, the regulation of 3.2% of positively QS‐dependent genes and 33.1% of negatively QS‐dependent genes was independent of both PhcR and PhcQ.

Strain OE1‐1 produces and secretes 3‐OH MAME and senses the chemical through PhcS, activating QS (Kai et al., 2015; Ujita et al., 2019). PhcR (Clough et al., 1997) and PhcQ (Tang et al., 2020) are regulator proteins with a receiver domain without a DNA‐binding site. Single‐receiver‐domain proteins play roles as phospho‐relays or phospho‐sinks (Feldheim et al., 2018). Considering the results of the present study, we propose the following scenario to explain the Phc QS signalling cascade of strain OE1‐1. PhcQ may mainly contribute to the activation of PhcA required for the regulation of 96.8% and 66.9% of positively and negatively QS‐dependent genes, respectively, dependent on extracellular 3‐OH MAME content. PhcR may be partially involved in the activation of PhcA required for the regulation of 22.9% and 26.4% of positively and negatively QS‐dependent genes, respectively, along with PhcQ, dependent on extracellular 3‐OH MAME content. In addition, an unknown regulator may be involved in the activation of PhcA required for the regulation of 3.2% and 33.1% of positively and negatively QS‐dependent genes, respectively. In vitro experiments with purified proteins and phosphorylation assays could be required to demonstrate that PhcQ and PhcR can be phosphorylated by PhcS. The LysR‐type transcriptional regulator PhcA has a conserved structure with an N‐terminal DNA‐binding helix–turn–helix motif and a C‐terminal co‐inducer‐binding domain (Maddocks & Oyston, 2008). However, little information on the co‐inducer of PhcA is available. The roles of PhcR and PhcQ elucidated in this study in the regulation of QS‐dependent genes shed light on the activation mechanisms of PhcA function in the active state of QS.

## 4 EXPERIMENTAL PROCEDURES

### 4.1 Bacterial strains, plasmids, and growth conditions

We used the following *R*. *pseudosolanacearum* strains in this study: OE1‐1 (Kanda et al., 2003), the *phcA*‐deletion mutant Δ*phcA* (Mori et al., 2016), the *phcB*‐deletion mutant Δ*phcB* (Kai et al., 2015), and the *phcK*‐deletion mutant Δ*phcK* (Senuma et al., 2020). All *R*. *pseudosolanacearum* strains were routinely grown in quarter‐strength M63 medium at 30 °C. *Escherichia coli* strains were grown in Luria–Bertani medium (Hanahan, 1983) at 37 °C. Gentamycin (50 μg/ml) was used in selective media.

### 4.2 Phylogenetic analysis

Phylogenetic trees were constructed using deduced amino acid sequences of PhcB (464–468 amino acids), PhcA (347 amino acids), PhcR (368–374 amino acids), and PhcQ (336–337 amino acids) of RSSC strains. We constructed phylogenetic trees using the ClustalW program according to the neighbour‐joining method (Saitou & Nei, 1987), with genetic distances computed with Kimura's two‐parameter model (Kimura, 1980). The phylogenetic trees were drawn with TreeView.

### 4.3 Generation of the *phcR*‐deletion and *phcQ*‐deletion mutants

The oligonucleotide primers used for construction of recombinant plasmids are listed in Table S6. The fragments delta‐R‐1 and delta‐R‐2 for generation of the Δ*phcR* mutant and the fragments delta‐Q‐1 and delta‐Q‐2 for generation of the Δ*phcQ* mutant were amplified by PCR from the genomic DNA of strain OE1‐1. The fragments delta‐R and delta‐Q amplified using delta‐R‐1 and delta‐R‐2 were ligated into a pK18mobsacB vector (Kvitko & Collmer, 2011) to produce recombinant plasmids pdelta‐phcR and pdelta‐phcQ, respectively. Each plasmid was electroporated into OE1‐1 competent cells, which were prepared as previously described by Mori et al. (2016). Kanamycin‐sensitive, sucrose‐resistant recombinants Δ*phcR* and Δ*phcQ* were then selected.

### 4.4 Generation of a *phcQ*‐deletion mutant transformed with native *phcQ*

Using the oligonucleotide primers listed in Table S5, the fragments souho‐Q‐1 and souho‐Q‐2 were amplified by PCR from the genomic DNA of strain OE1‐1. The fragment souho‐Q amplified using souho‐Q‐1 and souho‐Q‐2 was ligated into a pUC18‐mini‐Tn*7*T‐Gm vector (Choi et al., 2005) to produce psouhoQ. This plasmid was electroporated into Δ*phcQ* competent cells with a T7 transposase expression vector pTNS2 (Choi et al., 2005). Finally, a gentamycin‐resistant transformant, *phc*Q‐comp, was selected.

### 4.5 Transcriptome analysis based on RNA‐seq

Total RNA was extracted from *R*. *pseudosolanacearum* strains grown in quarter‐strength M63 until OD_600_ = 0.3 with a High Pure RNA Isolation kit (Roche Diagnostics). Ribosomal RNA was eliminated from the extracted total RNA using a Ribo‐Zero rRNA Removal kit (gram‐negative bacteria; Illumina) as previously described (Hayashi et al., 2019a). Oriented, paired‐end RNA‐seq (2 × 100 bp) was performed on an Illumina HiSeq 2000 system. The generated reads were trimmed with Cutadapt v. 1.1 (http://code.google.com/p/cutadapt/) and Trimmomatic v. 0.32 (http://www.usadellab.org/cms/?page=trimmomatic) and then mapped with the TopHat program v. 2.0.10 (http://tophat.cbcb.umd.edu/). Three independent biological replicates were carried out per strain.

### 4.6 Differential gene expression analysis

Statistical analysis of the RNA‐seq data was performed in the R environment. Genes with zero counts in at least one OE1‐1 sample were excluded. RNA‐seq read counts of the remaining genes were normalized using the function calcNormFactors (trimmed mean of M value normalization) in the package edgeR (Robinson et al., 2010). To extract genes with significant expression changes, the following thresholds were applied: *q* < .05 and |log(FC)| ≥ 2. The false discovery rate (*q* value) was calculated in edgeR from Benjamini–Hochberg‐corrected *p* values. Hierarchical clustering of all normalized mean expression values based on their relative expression (counts per million) was performed using Cluster v. 3.0 software (de Hoon et al., 2004). The average value of three replicates per strain was used. Heatmaps were created in TreeView (Eisen et al., 1998).

### 4.7 RT‐qPCR

Total RNA was extracted from *R*. *pseudosolanacearum* strains grown in quarter‐strength M63 until OD_600_ = 0.01 or OD_600_ = 0.3 with a High Pure RNA Isolation kit. To determine the influence of exogenous 3‐OH MAME application on the regulation of QS‐dependent genes, total RNA was extracted from *R*. *pseudosolanacearum* strains grown in quarter‐strength M63 containing 0.1 μM 3‐OH MAME until OD_600_ = 0.3 with a High Pure RNA Isolation kit. An RT‐qPCR assay with gene‐specific primers (Table S7) was carried out using the SYBR GreenER qPCR Reagent system (Invitrogen) on a 7300 Real‐Time PCR system (Applied Biosystems) as previously described (Hayashi et al., 2019a). All values were normalized against the expression level of *rpoD*, which was used as an internal standard for each cDNA sample. No significant differences in *rpoD* expression levels were observed among *R*. *pseudosolanacearum* strains. Two replicate experiments conducted using independent samples with eight technical replicates per experiment produced similar results. Results of a single representative sample are provided.

### 4.8 QS‐dependent phenotypes

We examined the in vitro biofilm formation of *R*. *pseudosolanacearum* strains grown without shaking in quarter‐strength M63 as previously described (Mori et al., 2016). To determine the influence of exogenous 3‐OH MAME application on biofilm formation, *R*. *pseudosolanacearum* strains were grown without shaking in quarter‐strength M63 containing 0.1 μM 3‐OH MAME as previously described (Senuma et al., 2020). The biofilm formation was quantified on the basis of the absorbance at 550 nm (A_550_). The resulting value was normalized according to the number of cells (OD_600_). Three replicate experiments conducted using independent samples with seven technical replicates per experiment produced similar results. The results of a representative experiment are shown.

EPS I production by *R*. *pseudosolanacearum* cells grown on quarter‐strength M63 solidified with 1.5% agar was quantitatively analysed in an enzyme‐linked immunosorbent assay (Agdia) as previously described (Mori et al., 2016). To determine the influence of exogenous 3‐OH MAME application on EPS I production, *R*. *pseudosolanacearum* strains were grown on quarter‐strength M63 solidified with 1.5% agar containing 0.1 μM 3‐OH MAME. EPS I production was quantified according to the absorbance at 650 nm (A_650_). Three replicate experiments conducted using independent samples with five technical replicates per experiment produced similar results. The results of a representative experiment are shown.

To assay ralfuranone A production, *R*. *pseudosolanacearum* strains were incubated in B medium at 30 °C for 4–6 hr and then diluted to an OD_600_ of 0.05 in new MGRL medium containing 3% sucrose (Ujita et al., 2019). The cell suspensions (2 ml) were incubated in 15‐ml test tubes at 30 °C with shaking for 2 days. Following growth, ralfuranone A was extracted from the culture supernatants with MonoSpin C18 (GL Science) according to the manufacturer's instructions. The methanol eluates from the MonoSpin C18 extraction were analysed for ralfuranones by liquid chromatography–mass spectrometry (LC‐MS) using an InertSustain C18 column (150 × 2.1 mm, 3 μm) under the following conditions: eluent = 20%–95% MeCN in 0.1% aqueous formic acid (0–24 min) and 95% MeCN (24–30 min); column temperature, 40 °C; flow rate, 200 μl/min; injection volume, 5 μl. The experiment was conducted three times using independently prepared samples.

For the swimming motility assay, overnight cultures of *R*. *pseudosolanacearum* strains were washed with distilled water and then diluted to a cell density of 5 × 10^5^ cfu/ml, and 5‐μl aliquots of cell suspensions were added to the centre of plates containing quarter‐strength M63 medium solidified with 0.25% agar. The swimming‐area diameters of *R*. *pseudosolanacearum* strains were measured at 48 hr postinoculation (Mori et al., 2018). To determine the influence of exogenous 3‐OH MAME application on swimming motility, 5‐μl aliquots of cell suspensions without or with 3‐OH MAME at a concentration of 0.1 μM were added to the centre of plates containing quarter‐strength M63 medium solidified with 0.25% agar. The swimming‐area diameters of *R*. *pseudosolanacearum* strains were measured at 24 hr postinoculation. Three replicate experiments conducted using independent samples with five technical replicates per experiment produced similar results. The results of a representative experiment are shown.

### 4.9 3‐OH MAME contents produced by *R. pseudosolanacearum* strains

Strains of *R. pseudosolanacearum* grown in B medium at 30 °C for 4–6 hr were diluted to an OD_600_ of 1.0 with new medium. The cell suspension at a volume of 50 μl was pipetted onto a BG agar plate (90 mm, 25 ml; Kai et al., 2015), and the plate was incubated for 24 hr at 30 °C. The BG agar was cut into small pieces and soaked in ethyl acetate at a volume of 50 ml for 2 hr twice. The combined extracts were dried over Na_2_SO_4_ and concentrated. The residue was dissolved in acetonitrile (100 ml) and subjected to LC‐MS analysis. LC‐MS data were recorded with an LCMS‐2020 (Shimadzu) and an InertSustain C18 column (150 mm ×2.1 mm, 3 μm particle size; GL Sciences). The conditions used were as follows: injection, 5 ml; solvent A, 0.1% formic acid; solvent B, acetonitrile containing 0.1% formic acid; gradient, 20%–95% B (0–24 min), 95% B (24–30 min); flow rate, 200 ml/min; detection, positive SIM mode; data reflect four replicates each.

### 4.10 Siderophore‐mediated iron acquisition activity

The siderophore‐mediated iron acquisition activity of *R*. *pseudosolanacearum* strains was analysed using a method modified from Wali et al. (2015). *R. pseudosolanacearum* strains were incubated in PY medium (5 g/L polypeptone and 2 g/L yeast extract) for 18 hr at 30 °C and adjusted to a concentration of 2 × 10^9^ cfu/ml with 0.1 M PIPES buffer (pH 6.5). After 6 hr of incubation, each culture was filtered through a 0.2‐μm pore filter. Next, 100 µl of a culture, or PIPES buffer alone as a reference, was added to 100 μl of chromazurol S (CAS) solution (2.4 mM hexadecyl‐trimethyl ammonium bromide, 0.06 mM FeCl_3_, 0.6 mM HCl, 0.6 mM CAS in PIPES buffer). Absorbance at 630 nm (A_630_) was measured after incubation for 30 min at 30 °C. The activity of the siderophore was calculated by subtracting the absorbance value of the reference from the total absorbance. Three replicate experiments conducted using independent samples with eight technical replicates per experiment produced similar results. Results of a single representative sample are provided.

### 4.11 Virulence assays

Eight‐week‐old tomato plants (*Solanum lycopersicum* ‘Ohgata‐Fukuju’) were inoculated with *R*. *pseudosolanacearum* strains (10^8^ cfu/ml) using a root‐dip inoculation procedure as previously described (Hayashi et al., 2019a).

We assessed populations of *R*. *pseudosolanacearum* strains in inoculated tomato roots according to their observed growth on Hara–Ono medium as described by Hayashi et al. (2019a). For each bacterial strain, three replicate experiments conducted using independent samples with five technical replicates per experiment produced similar results. Results of a single representative sample are provided.

The behaviour of *R*. *pseudosolanacearum* strains in tomato plants inoculated by the root‐dip method was assessed as described by Hayashi et al. (2019a). A sample from each cut site (Figure 3b) was pressed onto Hara–Ono medium. Twelve plants were analysed per trial, and each assay comprised five successive trials.

Plants were monitored daily for wilting symptoms, which were rated according to the following disease index scale: 0, no wilting; 1, 1%–25% wilting; 2, 26%–50% wilting; 3, 51%–75% wilting; 4, 76%–99%; and 5, dead. For each bacterial strain, three replicate experiments conducted using independent samples with 12 technical replicates per experiment produced similar results. Results of a single representative sample are provided.

### 4.12 Statistical analysis

The means of all assays were analysed for significant differences between *R*. *pseudosolanacearum* strains by Student's *t* test in Microsoft Excel.

## CONFLICT OF INTEREST

The authors declare that they have no conflicts of interest.

## Supporting information


**FIGURE S1** Phylogenetic trees of *Ralstonia solanacearum* species complex isolates based on the deduced amino acid sequences of PhcR and PhcQ. The scale bar indicates the genetic distance. The number provided in each node corresponds to the phylogenetic group listed in Table 1. The number provided next to each node indicates the bootstrap values of 1,000 replicates

**FIGURE S2** RNA‐sequencing transcriptome analysis of PhcR‐regulated or PhcQ‐regulated genes of *Ralstonia pseudosolanacearum* strains OE1‐1, Δ*phcB*, Δ*phcA*, Δ*phcR*, and Δ*phcQ* grown in quarter‐strength M63 medium until OD_600_ = 0.3. Three independent biological replicates were carried out per strain. (a, c) The numbers of genes exhibiting log_2_(fold change) ≤ −2 in Δ*phcB*, Δ*phcA*, Δ*phcR* (a), or Δ*phcQ* (c) mutants relative to their expression levels in strain OE1‐1 (*q* < .05). (b, d) The numbers of genes exhibiting log_2_(fold change) ≥ 2 in Δ*phcB*, Δ*phcA*, Δ*phcR* (b), or Δ*phcQ* (d) mutants relative to their expression levels in strain OE1‐1 (*q* < .05)

**TABLE S1** Phylotypes and phylogenetic types as determined using deduced amino acid sequences of PhcR and PhcQ of *Ralstonia solanacearum* species complex strains

**TABLE S2** RNA‐sequencing data for transcripts from *Ralstonia pseudosolanacearum* strains OE1‐1, Δ*phcB*, Δ*phcK*, Δ*phcA*, Δ*phcR*, and Δ*phcQ* grown in quarter‐strength M63 medium, and predicted functions of proteins encoded by the genes

**TABLE S3** RNA‐sequencing data for transcripts of PhcR‐regulated genes in *Ralstonia pseudosolanacearum* strains OE1‐1, Δ*phcB*, Δ*phcK*, Δ*phcA*, Δ*phcR*, and Δ*phcQ* grown in quarter‐strength M63 medium, and predicted functions of proteins encoded by the genes

**TABLE S4** RNA‐sequencing data for transcripts of PhcQ‐regulated genes in *Ralstonia pseudosolanacearum* strains OE1‐1, Δ*phcB*, Δ*phcK*, Δ*phcA*, Δ*phcR*, and Δ*phcQ* grown in quarter‐strength M63 medium, and predicted functions of proteins encoded by the genes

**TABLE S5** RNA‐sequencing data for transcripts of genes involved in siderophore‐mediated iron acquisition in *Ralstonia pseudosolanacearum* strains OE1‐1, Δ*phcB*, Δ*phcK*, Δ*phcA*, Δ*phcR*, and Δ*phcQ* grown in quarter‐strength M63 medium, and predicted functions of proteins encoded by the genes

**TABLE S6** Primers used in the generation of the *phcR‐* and *phcQ‐*deletion mutants from *Ralstonia pseudosolanacearum* strain OE1‐1 and the *phcQ*‐deletion mutant transformed with native *phcQ*

**TABLE S7** Primers used in the quantitative real‐time PCR assays

## Data Availability

The data that support the findings of this study are available from the corresponding author upon reasonable request.

## References

[mpp13124-bib-0001] Araud‐Razou, I. , Vasse, J. , Montrozier, H. , Etchebar, C. & Trigalet, A. (1998) Detection and visualization of the major acidic extracellular polysaccharide of *Ralstonia solanacearum* and its role in tomato root infection and vascular colonization. European Journal of Plant Pathology, 104, 795–809.

[mpp13124-bib-0002] Bhatt, G. & Denny, T.P. (2004) *Ralstonia solanacearum* iron scavenging by the siderophore staphyloferrin B is controlled by PhcA, the global virulence regulator. Journal of Bacteriology, 186, 7896–7904. 15547261 10.1128/JB.186.23.7896-7904.2004 PMC529077

[mpp13124-bib-0003] Castillo, J.A. & Agathos, S.N. (2019) A genome‐wide scan for genes under balancing selection in the plant pathogen *Ralstonia solanacearum* . BMC Ecology and Evolution, 19, 123. 10.1186/s12862-019-1456-6 PMC6580516 31208326

[mpp13124-bib-0004] Charlesworth, D. (2006) Balancing selection and its effects on sequences in nearby genome regions. PLoS Genetics, 2, e64. 16683038 10.1371/journal.pgen.0020064 PMC1449905

[mpp13124-bib-0005] Choi, K.‐H. , Gaynor, J.B. , White, K.G. , Lopez, C. , Bosio, C.M. , Karkhoff‐Schweizer, R.R. et al. (2005) A Tn*7*‐based broad‐range bacterial cloning and expression system. Nature Methods, 2, 443–448. 15908923 10.1038/nmeth765

[mpp13124-bib-0006] Clough, S.J. , Lee, K.E. , Schell, M.A. & Denny, T.P. (1997) A two‐component system in *Ralstonia* (*Pseudomonas*) *solanacearum* modulates production of PhcA‐regulated virulence factors in response to 3‐hydroxypalmitic acid methyl ester. Journal of Bacteriology, 179, 3639–3648. 9171411 10.1128/jb.179.11.3639-3648.1997 PMC179159

[mpp13124-bib-0008] Eisen, M.B. , Spellman, P.T. , Brown, P.O. & Botstein, D. (1998) Cluster analysis and display of genome‐wide expression patterns. Proceedings of the National Academic of Sciences of the United States of America, 95, 14863–14868. 10.1073/pnas.95.25.14863 PMC24541 9843981

[mpp13124-bib-0009] Fegan, M. & Prior, P. (2006) Diverse members of the *Ralstonia solanacearum* species complex cause bacterial wilts of banana. Australasian Plant Pathology, 35, 93–101.

[mpp13124-bib-0010] Feldheim, Y.S. , Zusman, T. , Kapach, A. & Segal, G. (2018) The single‐domain response regulator LerC functions as a connector protein in the *Legionella pneumophila* effectors regulatory network. Molecular Microbiology, 110, 741–760. 30105799 10.1111/mmi.14101

[mpp13124-bib-0011] Flavier, A.B. , Clough, S.J. , Schell, M.A. & Denny, T.P. (1997) Identification of 3‐hydroxypalmitic acid methyl ester as a novel autoregulator controlling virulence in *Ralstonia solanacearum* . Molecular Microbiology, 26, 251–259. 9383151 10.1046/j.1365-2958.1997.5661945.x

[mpp13124-bib-0012] Galloway, W.R. , Hodgkinson, J.T. , Bowden, S.D. , Welch, M. & Spring, D.R. (2011) Quorum sensing in Gram‐negative bacteria: small‐molecule modulation of AHL and AI‐2 quorum sensing pathways. Chemical Reviews, 111, 28–67. 21182299 10.1021/cr100109t

[mpp13124-bib-0013] Genin, S. & Denny, T.P. (2012) Pathogenomics of the *Ralstonia solanacearum* species complex. Annual Review of Phytopathology, 50, 67–89. 10.1146/annurev-phyto-081211-173000 22559068

[mpp13124-bib-0014] Guidot, A. , Jiang, W. , Ferdy, J.‐B. , Thébaud, C. , Barberis, P. , Gouzy, J. et al. (2014) Multihost experimental evolution of the pathogen *Ralstonia solanacearum* unveils genes involved inadaptation to plants. Molecular Biology and Evolution, 31, 2913–2928. 25086002 10.1093/molbev/msu229

[mpp13124-bib-0015] Ham, J.H. (2013) Intercellular and intracellular signaling systems that globally control the expression of virulence genes in plant pathogenic bacteria. Molecular Plant Pathology, 14, 308–322. 23186372 10.1111/mpp.12005 PMC6638695

[mpp13124-bib-0016] Hanahan, D. (1983) Studies on transformation of *Escherichia coli* with plasmids. Journal of Molecular Biology, 166, 557–580. 6345791 10.1016/s0022-2836(83)80284-8

[mpp13124-bib-0017] Hayashi, K. , Kai, K. , Mori, Y. , Ishikawa, S. , Ujita, Y. , Ohnishi, K. et al. (2019a) Contribution of a lectin, LecM, to the quorum sensing signaling pathway of *Ralstonia solanacearum* strain OE1‐1. Molecular Plant Pathology, 20, 334–345. 30312504 10.1111/mpp.12757 PMC6637872

[mpp13124-bib-0018] Hayashi, K. , Senuma, W. , Kai, K. , Takahashi, K. , Takemura, C. , Kawamoto, H. et al. (2019b) Major exopolysaccharide, EPS I, is associated with the feedback loop in the quorum sensing of *Ralstonia solanacearum* strain OE1‐1. Molecular Plant Pathology, 20, 1740–1747. 31560834 10.1111/mpp.12870 PMC6859485

[mpp13124-bib-0019] Hedrick, P.W. (2012) What is the evidence for heterozygote advantage selection? Trends in Ecology & Evolution, 27, 698–704. 22975220 10.1016/j.tree.2012.08.012

[mpp13124-bib-0020] Hikichi, Y. , Mori, Y. , Ishikawa, S. , Hayashi, K. , Ohnishi, K. , Kiba, A. et al. (2017) Regulation involved in colonization of intercellular spaces of host plants in *Ralstonia solanacearum* . Frontiers in Plant Science, 8, 967. 28642776 10.3389/fpls.2017.00967 PMC5462968

[mpp13124-bib-0007] de Hoon, M.J.L. , Imoto, S. , Nolan, J. & Miyano, S. (2004) Open source clustering software. Bioinformatics, 20, 1453–1454. 14871861 10.1093/bioinformatics/bth078

[mpp13124-bib-0021] Huang, J. & Schell, M. (1995) Molecular characterization of the *eps* gene cluster of *Pseudomonas solanacearum* and its transcriptional regulation at a single promoter. Molecular Microbiology, 16, 977–989. 7476194 10.1111/j.1365-2958.1995.tb02323.x

[mpp13124-bib-0022] Kai, K. , Ohnishi, H. , Mori, Y. , Kiba, A. , Ohnishi, K. & Hikichi, Y. (2014) Involvement of ralfuranone production in the virulence of *Ralstonia solanacearum* OE1‐1. ChemBioChem, 15, 2590–2597. 25250839 10.1002/cbic.201402404

[mpp13124-bib-0023] Kai, K. , Ohnishi, H. , Shimatani, M. , Ishikawa, S. , Mori, Y. , Kiba, A. et al. (2015) Methyl 3‐hydroxymyristate, a diffusible signal mediating *phc* quorum sensing in *Ralstonia solanacearum* . ChemBioChem, 16, 2309–2318. 26360813 10.1002/cbic.201500456

[mpp13124-bib-0024] Kanda, A. , Yasukohchi, M. , Ohnishi, K. , Kiba, A. , Okuno, T. & Hikichi, Y. (2003) Ectopic expression of *Ralstonia solanacearum* effector protein PopA early in invasion results in loss of virulence. Molecular Plant‐Microbe Interactions, 16, 447–455. 12744516 10.1094/MPMI.2003.16.5.447

[mpp13124-bib-0025] Kiba, A. , Nakano, M. , Hosokawa, M. , Galis, I. , Nakatani, H. , Shinya, T. et al. (2020) Phosphatidylinositol‐phospholipase C2 regulates pattern‐triggered immunity in *Nicotiana benthamiana* . Journal of Experimental Botany, 71, 5027–5038. 32412590 10.1093/jxb/eraa233 PMC7410187

[mpp13124-bib-0026] Kiba, A. , Nakano, M. , Ohnishi, K. & Hikichi, Y. (2018) The SEC14 phospholipid transfer protein regulates pathogen‐associated molecular pattern‐triggered immunity in *Nicotiana benthamiana* . Plant Physiology and Biochemistry, 125, 212–218. 29475087 10.1016/j.plaphy.2018.02.002

[mpp13124-bib-0027] Kimura, M. (1980) A simple method for estimating evolutionary rates of base substitutions through comparative studies of nucleotide sequences. Journal of Molecular Evolution, 16, 111–120. 7463489 10.1007/BF01731581

[mpp13124-bib-0028] Kreutzer, M.F. , Kage, H. , Gebhardt, P. , Wackler, B. , Saluz, H.P. , Hoffmeister, D. et al. (2011) Biosynthesis of a complex yersiniabactin‐like natural product via the mic locus in phytopathogen *Ralstonia solanacearum* . Applied and Environmental Microbiology, 77, 6117–6124. 21724891 10.1128/AEM.05198-11 PMC3165373

[mpp13124-bib-0029] Kvitko, B.H. & Collmer, A. (2011) Construction of *Pseudomonas syringae* pv. *tomato* DC3000 mutant and polymutant strains. Methods in Molecular Biology, 712, 109–128. 21359804 10.1007/978-1-61737-998-7_10

[mpp13124-bib-0030] Maddocks, S.E. & Oyston, P.C.F. (2008) Structure and function of the LysR‐type transcriptional regulator (LTTR) family protein. Microbiology, 154, 3609–3623. 19047729 10.1099/mic.0.2008/022772-0

[mpp13124-bib-0031] Mansfield, J. , Genin, S. , Magori, S. , Citovsky, V. , Sriariyanum, M. , Ronald, P. et al. (2012) Top 10 plant pathogenic bacteria in molecular plant pathology. Molecular Plant Pathology, 13, 614–629. 22672649 10.1111/j.1364-3703.2012.00804.x PMC6638704

[mpp13124-bib-0032] Mori, Y. , Inoue, K. , Ikeda, K. , Nakayashiki, H. , Higashimoto, C. , Ohnishi, K. et al. (2016) The vascular plant‐pathogenic bacterium *Ralstonia solanacearum* produces biofilms required for its virulence on the surfaces of tomato cells adjacent to intercellular spaces. Molecular Plant Pathology, 17, 890–902. 26609568 10.1111/mpp.12335 PMC6638453

[mpp13124-bib-0033] Mori, Y. , Ohnishi, H. , Shimatani, M. , Morikawa, Y. , Ishikawa, S. , Ohnishi, K. et al. (2018) Involvement of ralfuranones in the quorum sensing signalling pathway and virulence of *Ralstonia solanacearum* strain OE1‐1. Molecular Plant Pathology, 19, 454–463. 28116815 10.1111/mpp.12537 PMC6638173

[mpp13124-bib-0034] Nakano, M. , Nishihara, M. , Yoshioka, H. , Takahashi, H. , Sawasaki, T. , Ohnishi, K. et al. (2013) Suppression of DS1 phosphatidic acid phosphatase confirms resistance to *Ralstonia solanacearum* in *Nicotiana benthamiana* . PLoS One, 8, e75124. 24073238 10.1371/journal.pone.0075124 PMC3779229

[mpp13124-bib-0035] Pauly, J. , Spiteller, D. , Linz, J. , Jacobs, J. , Allen, C. , Nett, M. et al. (2013) Ralfuranone thioether production by the plant pathogen *Ralstonia solanacearum* . ChemBioChem, 14, 2169–2178. 24106142 10.1002/cbic.201300364

[mpp13124-bib-0037] Robinson, M.D. , McCarthy, D.J. & Smyth, G.K. (2010) edgeR: A bioconductor package for differential expression analysis of digital gene expression data. Bioinformatics, 26, 139–140. 19910308 10.1093/bioinformatics/btp616 PMC2796818

[mpp13124-bib-0038] Rutherford, S.T. & Bassler, B.L. (2012) Bacterial quorum sensing: its role in virulence and possibilities for its control. Cold Spring Harbor Perspectives in Medicine, 1, a012427. 10.1101/cshperspect.a012427 PMC3543102 23125205

[mpp13124-bib-0039] Safni, I. , Cleenwerck, I. , De Vos, P. , Fegan, M. , Sly, L. & Kappler, U. (2014) Polyphasic taxonomic revision of the *Ralstonia solanacearum* species complex: proposal to emend the descriptions of *Ralstonia solanacearum* and *Ralstonia syzygii* and reclassify current *R*. *syzygii* strains as *Ralstonia syzygii* subsp. *syzygii* subsp. nov., *R*. *solanacearum* phylotype IV strains as *Ralstonia syzygii* subsp. *indonesiensis* subsp. nov., banana blood disease bacterium strains as *Ralstonia syzygii* subsp. *celebesensis* subsp. nov. and *R*. *solanacearum* phylotype I and III strains as *Ralstonia pseudosolanacearum* sp. nov. International Journal of Systematic and Evolutionary Microbiology, 64, 3087–3103. 24944341 10.1099/ijs.0.066712-0

[mpp13124-bib-0040] Saitou, N. & Nei, M. (1987) The neighbor‐joining method: a new method for reconstructing phylogenetic trees. Molecular Biology and Evolution, 4, 406–425. 3447015 10.1093/oxfordjournals.molbev.a040454

[mpp13124-bib-0041] Salanoubat, M. , Genin, S. , Artiguenave, F. , Gouzy, J. , Mangenot, S. , Arlat, M. et al. (2002) Genome sequence of the plant pathogen *Ralstonia solanacearum* . Nature, 415, 497–502. 11823852 10.1038/415497a

[mpp13124-bib-0042] Schell, M.A. (2000) Control of virulence and pathogenicity genes of *Ralstonia solanacearum* by an elaborate sensory network. Annual Review of Phytopathology, 38, 263–292. 10.1146/annurev.phyto.38.1.263 11701844

[mpp13124-bib-0043] Senuma, W. , Takemura, C. , Hayashi, K. , Ishikawa, S. , Kiba, A. , Ohnishi, K. et al. (2020) The putative sensor histidine kinase PhcK is required for the full expression of *phcA* encoding the global transcriptional regulator to drive the quorum sensing circuit of *Ralstonia solanacearum* strain OE1‐1. Molecular Plant Pathology, 21, 1591–1605. 33025726 10.1111/mpp.12998 PMC7694676

[mpp13124-bib-0044] Stoeckel, S. , Klein, E.K. , Oddou‐Muratorio, S. , Much, B. & Mariette, S. (2012) Microevolution of s‐allele frequencies in wild cherry populations: respective impacts of negative frequency dependent selection and genetic drift: selection versus genetic drift at the s‐locus between two generations. Evolution, 66, 486–504. 22276543 10.1111/j.1558-5646.2011.01457.x

[mpp13124-bib-0045] Tang, M. , Bouchez, O. , Cruveiller, S. , Masson‐Boivin, C. & Capela, D. (2020) Modulation of quorum sensing as an adaptation to nodule cell infection during experimental evolution of legume symbionts. mBio, 1, e03129‐19. 10.1128/mBio.03129-19 PMC6989110 31992622

[mpp13124-bib-0046] Tans‐Kersten, J. , Huang, H. & Allen, C. (2001) *Ralstonia solanacearum* needs motility for invasive virulence on tomato. Journal of Bacteriology, 183, 3597–3605. 11371523 10.1128/JB.183.12.3597-3605.2001 PMC95236

[mpp13124-bib-0047] Ujita, Y. , Sakata, M. , Yoshihara, A. , Hikichi, Y. & Kai, K. (2019) Signal production and response specificity in the phc quorum sensing systems of *Ralstonia solanacearum* species complex. ACS Chemical Biology, 14, 2243–2251. 31513382 10.1021/acschembio.9b00553

[mpp13124-bib-0048] Vasse, J. , Frey, P. & Trigalet, A. (1995) Microscopic studies of intercellular infection and protoxylem invasion of tomato roots by *Pseudomonas solanacearum* . Molecular Plant‐Microbe Interactions, 8, 241–251.

[mpp13124-bib-0049] Wackler, B. , Schneider, P. , Jacobs, J. , Pauly, J. , Allen, C. , Nett, M. et al. (2011) Ralfuranone biosynthesis in *Ralstonia solanacearum* suggests functional divergence in the quinone synthetase family of enzymes. Chemistry and Biology, 18, 354–360. 21439480 10.1016/j.chembiol.2011.01.010

[mpp13124-bib-0050] Wali, U.M. , Maenaka, R. , Mori, Y. , Ueno, D. , Kai, K. , Ohnishi, K. et al. (2015) Implication of limited iron acquisition of *Pseudomonas cichorii* strain SPC9018 in reduction of its virulence on eggplant. Journal of General Plant Pathology, 81, 136–141.

[mpp13124-bib-0051] Waters, C.M. & Bassler, B.L. (2005) Quorum sensing: cell‐to‐cell communication in bacteria. Annual Review of Cell and Developmental Biology, 21, 319–346. 10.1146/annurev.cellbio.21.012704.131001 16212498

