# Supplementary figures and images for: PhcQ mainly contributes to the regulation of quorum sensing‐dependent genes, in which PhcR is partially involved, in Ralstonia pseudosolanacearum strain OE1‐1
Source: Mol Plant Pathol. 2021 Aug 21;22(12):1538–52. doi: 10.1111/mpp.13124 (PMC8578825; doi:10.1111/mpp.13124)

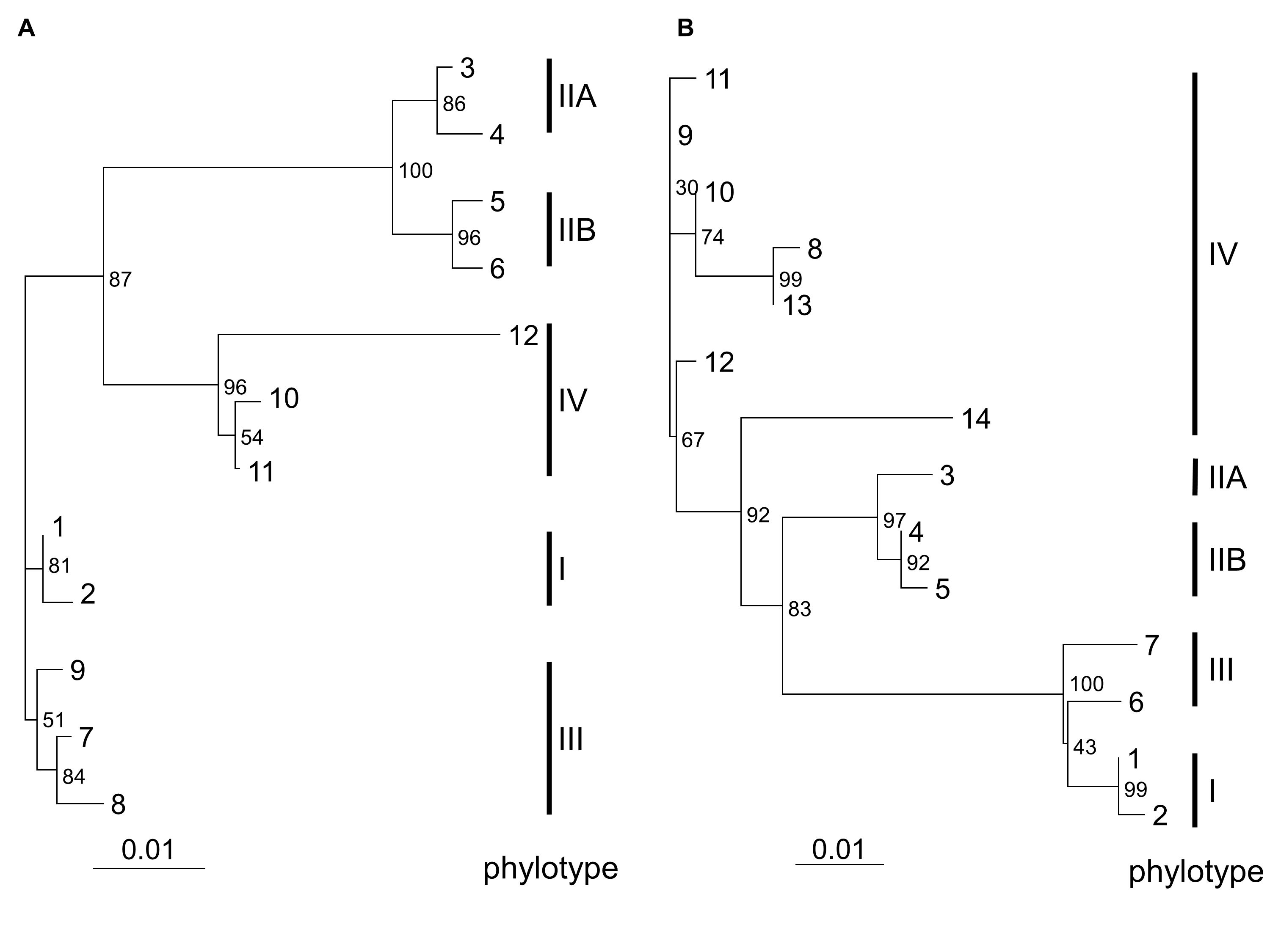

Supplement: Supplementary file 1 — FIGURE S1 Phylogenetic trees of Ralstonia solanacearum species complex isolates based on the deduced amino acid sequences of PhcR and PhcQ. The scale bar indicates the genetic distance. The number provided in each node corresponds to the phylogenetic group listed in Table 1. The number provided next to each node indicates the bootstrap values of 1,000 replicates [file MPP-22-1538-s009.tif]

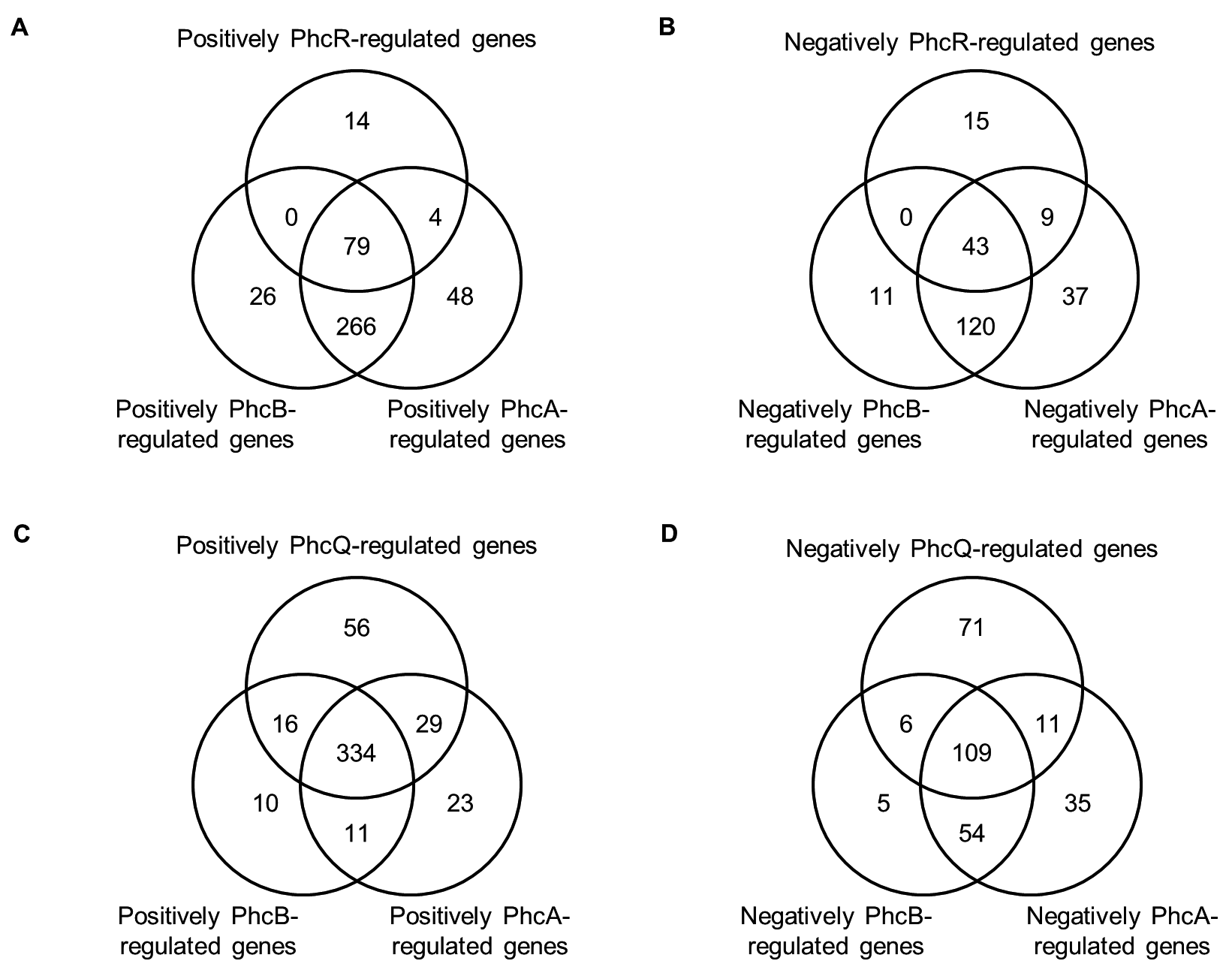

Supplement: Supplementary file 2 — FIGURE S2 RNA‐sequencing transcriptome analysis of PhcR‐regulated or PhcQ‐regulated genes of Ralstonia pseudosolanacearum strains OE1‐1, ΔphcB, ΔphcA, ΔphcR, and ΔphcQ grown in quarter‐strength M63 medium until OD600 = 0.3. Three independent biological replicates were carried out per strain. (a, c) The numbers of genes exhibiting log2(fold change) ≤ −2 in ΔphcB, ΔphcA, ΔphcR (a), or ΔphcQ (c) mutants relative to their expression levels in strain OE1‐1 (q < .05). (b, d) The numbers of genes exhibiting log2(fold change) ≥ 2 in ΔphcB, ΔphcA, ΔphcR (b), or ΔphcQ (d) mutants relative to their expression levels in strain OE1‐1 (q < .05) [file MPP-22-1538-s005.tif]
