# Supplementary material for: PhcQ mainly contributes to the regulation of quorum sensing‐dependent genes, in which PhcR is partially involved, in Ralstonia pseudosolanacearum strain OE1‐1
Source: Mol Plant Pathol. 2021 Aug 21;22(12):1538–52. doi: 10.1111/mpp.13124 (PMC8578825; doi:10.1111/mpp.13124)
Supplement: Supplementary file 8 — TABLE S6 Primers used in the generation of the phcR‐ and phcQ‐deletion mutants from Ralstonia pseudosolanacearum strain OE1‐1 and the phcQ‐deletion mutant transformed with native phcQ [file MPP-22-1538-s001.docx]

**Table S6** Primers used in the generation of the *phcR-* and *phcQ-* deletion mutants from *Ralstonia pseudosolanacearum* strain OE1-1 and the *phcQ*-deletion mutant transformed with native *phcQ*

| Constructed plasmid | fragment | primers | Sequence^a^ (5’-3’) | Restriction enzyme |
| --- | --- | --- | --- | --- |
| pdelta-phcR  pdelta-phcQ  psouhoQ | delta-R-1  delta-R-2  delta-Q-1  delta-Q-2  souho-Q-1  souho-Q-2 | delta-R-1-FW2  delta-R-1-RV2  delta-R-2-FW2  delta-R-2-RV2  delta-Q-1-FW2  delta-Q-1-RV2  delta-Q-2-FW2  delta-Q-2-RV2    souho-Q-1-FW  souho-Q-1-RV  souho-Q-2-FW  delta-Q-2-RV2 | cgggatccTGGCCGAAAAGAACGAGCAGCTG  CGGGGTTGGTCATGCTCGCTCCTATTCCGC  AGGAGCGAGCATGACCAACCCCGGGGAC  cccaagcttGCTTTCCTCGCCGACCAGTTC  cgggatccTCTCGCCCACGAACTGAACACC  TGGCCGGTCAGGTCATCCATCACTCCTCTTAG  TGATGGATGACCTGACCGGCCACCCCGCGG  cccaagcttCAACCGTTCTACGACCCGATGTG  ggggtaccTTCATCGAGTACTACCTGTACC  GGGGTTGGTCATGGT GCGAATTTGCCGGAGACTTC  CAAATTCGCACCATGACCAACCCCGGGGACAATG  cccaagcttCAACCGTTCTACGACCCGATGTG | BamHI  HindIII  BamHI  HindIII  KpnI  HindIII |

^a^ Small capital indicates restriction enzyme site.
