# Supplementary material for: PhcQ mainly contributes to the regulation of quorum sensing‐dependent genes, in which PhcR is partially involved, in Ralstonia pseudosolanacearum strain OE1‐1
Source: Mol Plant Pathol. 2021 Aug 21;22(12):1538–52. doi: 10.1111/mpp.13124 (PMC8578825; doi:10.1111/mpp.13124)
Supplement: Supplementary file 9 — TABLE S7 Primers used in the quantitative real‐time PCR assays [file MPP-22-1538-s008.docx]

**Table S7** Primers used in the quantitative real-time polymerase chain reaction assays

| Genes | primers | Nucleotide sequences |
| --- | --- | --- |
| *rpoD*  *epsB*  *fliC*  *ralA*  *lecM*  *phcB*  *phcA*  *phcK*  *phcR*  *phcQ* | rpoD-FW  rpoD-RV  epsB-FW  epsB-RV2  fliC-FW2  fliC-RV2  ralA-FW  ralA-RV  fml-FW2  fml-RV2  phcB-FW3-514  phcB-RV3-1011  phcA-FW5  phcA-RV5  phcK-FW  phcK-RV  phcR-FW  phcR-RV  phcQ-FW  phcQ-RV | 5ʹ-ATCGTCGAGCGCAACATCCC-3ʹ  5ʹ-AGATGGGAGTCGTCGTCGTCGTG-3ʹ  5ʹ-ATGGTCGAGCTGATGGATA-3ʹ  5ʹ-TGGAGCTGCTTGATCGTCTC-3ʹ  5ʹ-CAAACGCAAGGTATTCAGAACG-3ʹ  5ʹ-ATTGGAAGGTCGTCGAAGCCAC-3ʹ  5ʹ-GCCTGGGGATAAGGTTGTAC-3ʹ  5ʹ-CGTCAGTACGAAAACAGCG-3ʹ  5’-GTATTCACGCTTCCCGCCAACAC-3’  5’-ATGCCGTCGTTGTAGTCGTTGTC-3’  5ʹ-TACAAGATCAAGCACTACCTCGACTG-3ʹ  5ʹ-GTGCTGTACGCCATCCATCTC-3ʹ  5ʹ-ATGCGTTCCAATGAGCTGGAC-3ʹ  5ʹ-AGATCCTTCATCAGCGAGTTGAC-3ʹ  5ʹ-TGTCGATGTGGCTGCTGATC-3ʹ  5ʹ-CGTTGAACAGGAAATGCGGTTC-3ʹ  5ʹ-GCTGTCGACCTTCCTGAATTC-3ʹ  5ʹ-AAAACGCGGATCAGGTACGG-3ʹ  5ʹ-ATCCTGACCACCGCCTAC-3ʹ  5ʹ-CGACACCGCTAGGTACAG-3ʹ |
